# Hippocampal subregional texture features associated with Alzheimer’s disease severity and cognition

**DOI:** 10.1093/braincomms/fcag164

**Published:** 2026-05-06

**Authors:** Yuto Uchida, Kei Nishimaki, Hirohito Kan, Hitoshi Iyatomi, Kenichi Oishi

**Affiliations:** Department of Radiology and Radiological Science, Johns Hopkins University School of Medicine, Baltimore, MD 21205, USA; Department of Applied Informatics, Graduate School of Science and Engineering, Hosei University, Tokyo 184-8584, Japan; Department of Integrated Health Sciences, Nagoya University Graduate School of Medicine, Nagoya, Aichi 461-8673, Japan; Department of Applied Informatics, Graduate School of Science and Engineering, Hosei University, Tokyo 184-8584, Japan; Department of Radiology and Radiological Science, Johns Hopkins University School of Medicine, Baltimore, MD 21205, USA

**Keywords:** Alzheimer’s disease, hippocampal subregion, magnetic resonance imaging, radiomics, texture analysis

## Abstract

Alzheimer’s disease (AD) exhibits selective vulnerability in hippocampal subfields, where microstructural alterations precede overt atrophy. Conventional morphometric measures, such as volume or thickness, are limited in their ability to capture subtle textural heterogeneity that reflects underlying cytoarchitectural disorganization, particularly perforant path fibres within the subiculum. The objective of this study is to determine whether hippocampal subregional texture-based radiomic features can serve as sensitive markers of disease severity and cognitive impairment along the AD continuum. This retrospective, multicentre, cross-sectional study used data from the Alzheimer’s Disease Neuroimaging Initiative (ADNI). Participants who underwent coronal high-resolution T2-weighted ‘HighResHippo’ magnetic resonance imaging (MRI) and cerebrospinal fluid (CSF) biomarker assessment within 1 month of each other between June 2011 and May 2025 were included. Automatic Segmentation of Hippocampal Subfields was used to delineate cornu ammonis 1–3, dentate gyrus, subiculum, and other medial temporal cortices, followed by quality control to exclude mislabelled or artefact-degraded segmentations. After bias-field correction, intensity normalization, and voxel-resampling, first-order, shape, and texture features—including grey-level run-length matrix (GLRLM) metrics—were extracted. Participants were categorized according to cognitive status and CSF amyloid-β (Aβ) positivity. Associations between radiomic features and Montreal Cognitive Assessment (MoCA) scores as well as CSF tau concentrations were evaluated using multiple linear regression, adjusting for age, sex, education, and APOE ε4 status. Among 1264 screened participants, 241 met all inclusion criteria (mean age 73.4 ± 6.9 years; 54% women). GLRLM-based run entropy within the subiculum demonstrated a stepwise increase across the AD continuum (cognitively unimpaired-Aβ− < cognitively unimpaired-Aβ^+^ < mild cognitive impairment-Aβ^+^ < AD dementia-Aβ^+^). Orientation-specific analyses revealed that the superior–inferior component—aligned with the dominant fibre orientation of the subicular efferent pathway—showed the strongest associations with clinical and biomarker indices. Higher SI run entropy correlated with lower MoCA performance (standardized β = −0.710 [95% CI −1.416 to −0.038]) and higher CSF tau levels (standardized β = 2.307 [95% CI 0.767 to 3.848]) compared with other directional components. Radiomic texture features derived from *in vivo* high-resolution T2-weighted MRI of hippocampal subregions—particularly subicular GLRLM run entropy aligned with superior–inferior tract orientation—track disease severity and cognitive impairment along the AD continuum. These findings support texture-based imaging biomarkers as sensitive indicators of early microstructural alterations in AD.

## Teaser

Uchida *et al*. reported cross-sectional associations between subicular texture patterns, phosphorylated tau cerebrospinal fluid concentrations and cognitive performance, indicating that hippocampal texture analysis may provide complementary information about hippocampal microstructural heterogeneity along the Alzheimer’s disease continuum.

## Graphical Abstract

**Graphical Abstract:**
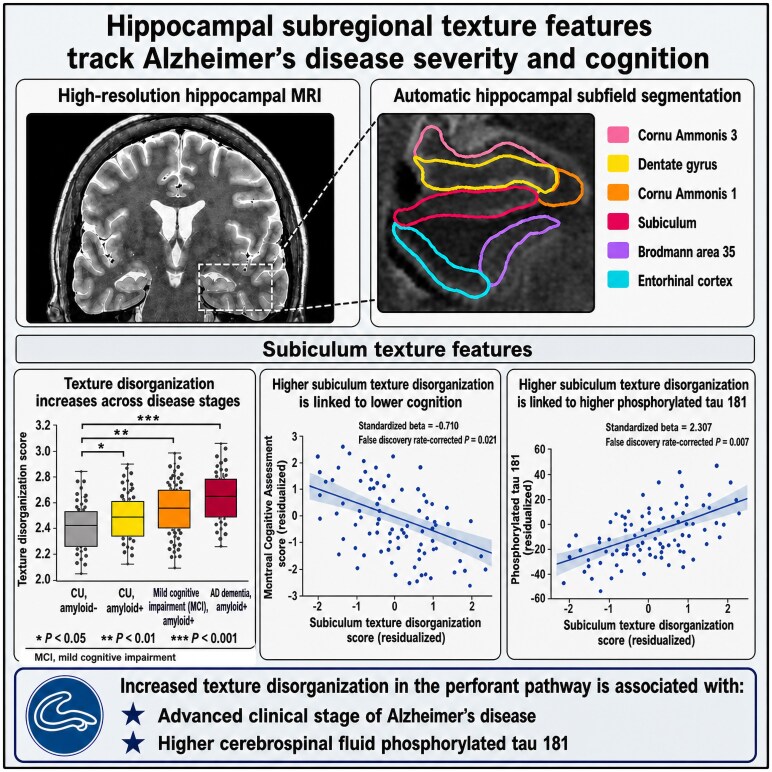

## Introduction

Alzheimer’s disease (AD) is a progressive neurodegenerative disorder characterized by memory loss and cognitive decline.^1^ One of the earliest brain structures affected in the context of AD pathology is the medial temporal lobe (MTL), including the hippocampus and entorhinal cortex (ERC),^2^ which plays a central role in memory encoding and spatial navigation.^3,4^ Neurodegeneration within the hippocampus occurs in a region-specific manner, with subfields such as the subiculum, cornu ammonis 1 (CA1) and dentate gyrus showing differential vulnerability.^5^ Accurate assessment of these subregions has therefore become a critical focus in the early diagnosis and monitoring of the AD continuum.^6-9^

In light of the accumulating evidence for the differential vulnerability of hippocampal subfields to AD pathophysiology,^9^ the Alzheimer’s Disease Neuroimaging Initiative (ADNI) has added a high-resolution coronal T2-weighted fast spin-echo sequence, called ‘HighResHippo.’^10^ This sequence is aligned along the hippocampal axis, providing 0.4 mm in-plane resolution for detailed visualization of the MTL subregions.^11^ Using this sequence, volumetry and thickness measurements of hippocampal subfields have demonstrated that specific regions, particularly the subiculum and ERC, are affected during the early stages of AD.^8,9^ However, these conventional morphometric measures primarily capture macroscopic structural changes and may overlook subtle microstructural alterations that precede visible atrophy.

Degeneration of perforant path fibres has recently emerged as a candidate for a highly sensitive and specific marker along the AD continuum.^12-15^ Anatomically, the perforant path represents the principal afferent pathway to the hippocampus, with axons originating in ERC and projecting through the subiculum to the dentate gyrus and CA1.^16,17^ Using postmortem brain tissues of the left MTL, submillimetre-resolution T2-weighted imaging and diffusion MRI have visualized degenerative changes in the perforant path fibres. These alterations were histologically evident in the subiculum, even in preclinical AD cases without measurable atrophy in the hippocampus or entorhinal cortex.^12,13^ We hypothesize that the selective degeneration of the perforant pathway, which runs perpendicular to the pial surface of the subiculum, results in a loss of randomness in signal intensity within this area.

In this study, we utilized HighResHippo T2-weighted MRI data from the ADNI database to extract radiomic features from hippocampal subregions at submillimetre resolutions. We compared the texture-based radiomic features across groups along the AD continuum and examined their associations with cognitive performance, aiming to assess whether the perforant path-related features can serve as non-invasive markers for the early detection of AD and cognitive decline.

## Materials and methods

### Study design and participants

The study was a retrospective, multicentre, observational cross-sectional study using the ADNI database (adni.loni.usc.edu). We included participants who had HighResHippo T2-weighted MRI and cerebrospinal fluid (CSF) biomarkers acquired within 1 month of each other between 13 June 2011 and 12 May 2025. When multiple eligible MRI scans were available for a single participant, the first MRI that met this criterion (i.e. the earliest scan within 1 month of the CSF collection) was selected to ensure consistency across participants for cross-sectional group comparisons. The Institutional Review Boards approved all ADNI procedures of participating institutions, and written informed consent was obtained from all participants or their legally authorized representatives in accordance with the Declaration of Helsinki.

### Neuropsychological assessments

Cognitive functions were comprehensively evaluated using the Clinical Dementia Rating (CDR), Mini-Mental State Examination (MMSE) and Montreal Cognitive Assessment (MoCA). The CDR provides a global score ranging from 0 to 3, reflecting overall dementia severity and also includes the Clinical Dementia Rating–Sum of Boxes (CDR-SB), which offers a more detailed assessment by summing scores across six functional and cognitive domains, with a total score ranging from 0 to 18.^18^ MMSE is a widely used screening test that primarily assesses orientation, calculation and language abilities, whereas MoCA additionally evaluates attention, visuospatial skills, and verbal and visual memory and has been validated as a more sensitive tool for the early detection of AD.^19^

### CSF biomarkers

CSF amyloid-β (Aβ) and tau biomarkers, such as phosphorylated tau at threonine 181 (p-tau181) and total tau (t-tau), were measured. Details of CSF biomarkers are described in Appendix S1 of Supplementary Materials.

### Classification of participants along the AD continuum

The use of the CSF Aβ biomarker classification, in combination with CDR, allowed for the stratification of study participants according to the AD continuum framework.^20^ Specifically, cognitively unimpaired (CU; i.e. CDR = 0) individuals were categorized into Aβ-negative (CU-Aβ^−^) and Aβ-positive (CU-Aβ^+^) subgroups based on their Aβ_1–42_ levels. In addition, participants with mild cognitive impairment (MCI; i.e. CDR = 0.5) or AD dementia (ADD; i.e. CDR ≥ 1) with Aβ^+^ were classified into the MCI-Aβ^+^ and ADD-Aβ^+^ groups, respectively. This classification scheme enabled both biologically and clinically informed subgrouping, thereby enhancing the specificity and interpretability of subsequent imaging and cognitive analyses.^21^

### MRI analyses

The HighResHippo MRI protocol is a high-resolution 2D T2-weighted turbo spin echo sequence (0.4 × 0.4 mm^2^, 2 mm thick) oriented perpendicular to the hippocampal axis,^10^ designed to visualize hippocampal subfields with fine anatomical detail (Appendix S2, Supplementary Fig. 1A). Using these images, the left hippocampal segmentation was performed with the Automatic Segmentation of Hippocampal Subfields (ASHS) v2.0.0, employing the PMC-T1T2 atlas (https://www.nitrc.org/projects/ashs).^8^ Segmentation was based on co-registered T1- and T2-weighted images, generating delineations of CA1–CA3, dentate gyrus, subiculum, ERC, and parahippocampal cortex (PHC), subdivided into Brodmann areas (BA) 35 and 36. Following automated segmentation, all outputs were visually inspected for quality assurance (Y.U., with 14 years of brain MRI research; Appendix S3, Supplementary Fig. 1B, Supplementary Fig. 2). Radiomic features were then extracted from ASHS-defined regions using PyRadiomics v3.0.1 after N4 bias correction and z-score normalization, including first-order statistical features (e.g. mean, dispersion, entropy, skewness and kurtosis), shape features (e.g. volume and thickness) and texture features derived from the grey-level run-length matrix (GLRLM), which characterizes the distribution of consecutive voxels with identical intensities.^22^ GLRLM-based run entropy, which reflects randomness of run-length patterns in uniform intensities, was further decomposed into superior–inferior (SI), left–right (LR) and anterior–posterior (AP) directions to capture structural anisotropy based on an *a priori* hypothesis for degeneration of perforant path fibres (Appendix S4, Supplementary Fig. 1C). All reported radiomic features adhere to the Image Biomarker Standardization Initiative nomenclature and reporting guidelines (Appendix S5).^23^

### Statistical analyses

For descriptive statistics, continuous variables were summarized as mean (standard deviation [SD]) or median (interquartile range [IQR]), depending on the normality of distribution. Categorical variables were reported as counts and percentages. Between-group comparisons along the AD continuum (e.g. CU-Aβ^−^, CU-Aβ^+^, MCI-Aβ^+^ and ADD-Aβ^+^) were assessed using the one-way analysis of variance or the Kruskal–Wallis test, as appropriate. When overall group differences were significant, post hoc pairwise comparisons (e.g. independent-sample t-tests or Mann–Whitney U tests) were performed, with *P* values adjusted for multiple comparisons using the Benjamini–Hochberg false discovery rate (FDR).

Since MRI data were acquired across multiple scanner vendors, potential batch effects were corrected using the ComBat harmonization.^24^ This empirical Bayes method was applied using scanner vendor (Siemens, GE and Philips) as the batch variable, with age, sex, years of education, APOE ε4 carrier status and disease stage included as biological covariates. The effectiveness of harmonization was further evaluated using pre- and post-harmonization feature distributions stratified by vendor and variance decomposition analyses (Supplementary Fig. 3).

To evaluate the associations between cognitive performance (e.g. MMSE, MoCA) or CSF tau levels (e.g. p-tau181, t-tau) and each radiomic feature, we constructed multiple linear regression models. In each model, the cognitive score or CSF tau level served as the dependent variable, and each radiomic feature was tested independently as the predictor to avoid multicollinearity, with adjustment for age, sex, years of education and APOE ε4 carrier status. Residualized values, obtained after regressing out these covariates, were used for visualization in partial regression plots. The Benjamini–Hochberg FDR correction was applied to control for multiple comparisons across the selected radiomic features and hippocampal subfields.

All statistical analyses were performed using Python version 3.10 (Python Software Foundation, Wilmington, DE). Reported results include 95% confidence intervals (CI) when applicable.

## Results

### Participant characteristics

**Figure 1:**
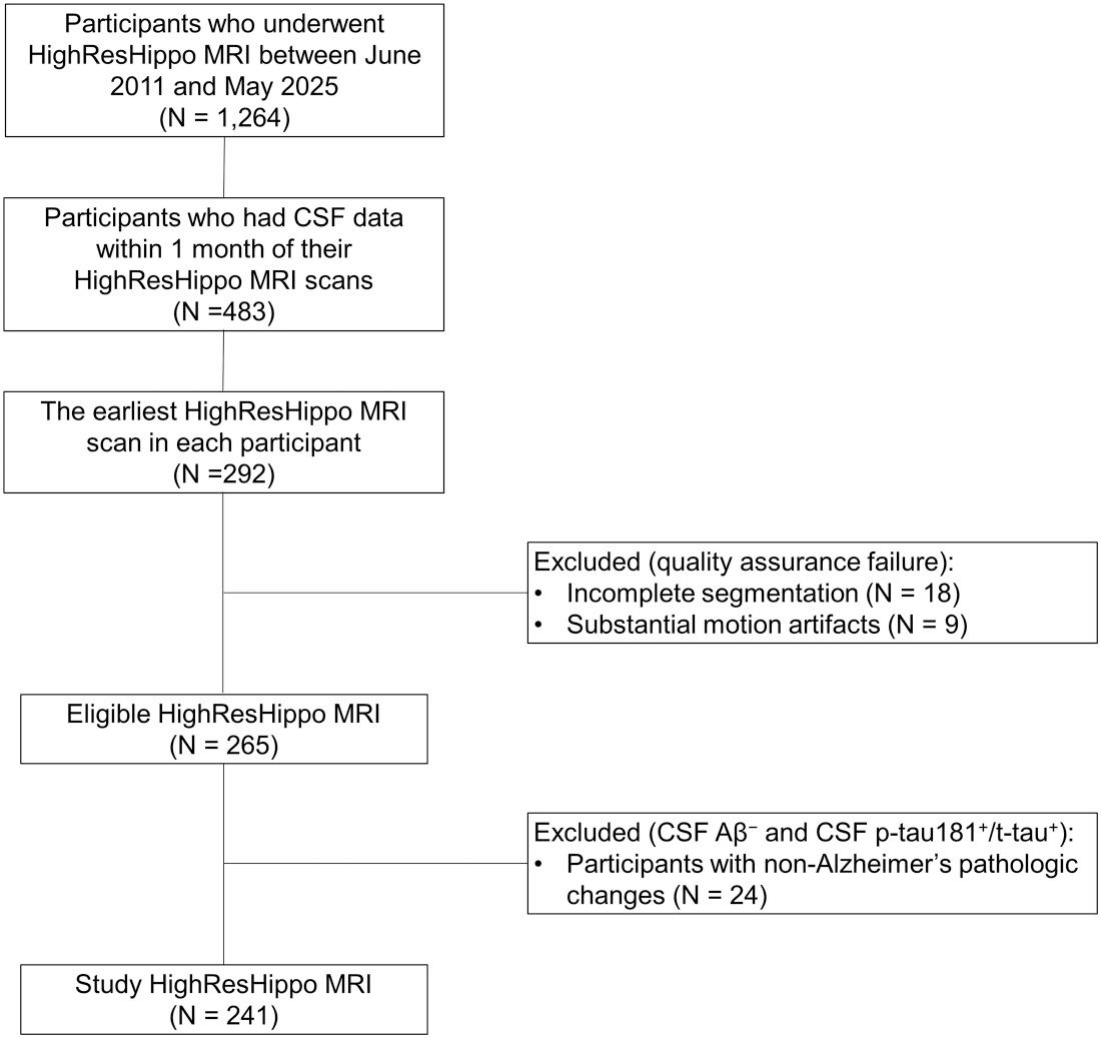
**Flowchart of the participant selection.** Of the 1264 participants who underwent the HighResHippo MRI between June 2011 and May 2025, 483 participants also underwent CSF examinations within 1 month of their MRI scans. When multiple eligible MRI scans were available for a single participant, the first MRI that met this criterion was selected. Among them, 18 HighResHippo MRIs with incomplete segmentation and nine with substantial motion artefacts were excluded. Further, 24 participants diagnosed with non-Alzheimer’s pathologic changes (i.e. CSF Aβ-negative and at least one of CSF p-tau181- or t-tau-positive) were excluded, resulting in 241 HighResHippo MRIs in the present study.

The clinical characteristics of study participants are summarized in Table 1. Overall, 241 participants (mean age at the baseline MRI scans [SD], 73.4 [6.9] years; 131 women [54%]) were matched to the inclusion criteria of this study (Fig. 1), comprising 76 CU-Aβ^−^, 45 CU-Aβ^+^, 89 MCI-Aβ^+^ and 31 ADD-Aβ^+^. No significant differences were observed among the groups in terms of age, sex or years of education. The prevalence of the *APOE* ɛ4 allele was less than 10% in CU-Aβ^−^, which was comparable to the general population.^25^ In contrast, the prevalence exceeded 30% in the Aβ^+^ groups, reaching over 60% in ADD-Aβ^+^. For neuropsychological assessments, CDR/CDR-SB, MMSE and MoCA scores showed progressive cognitive decline along the AD continuum. Similarly, for the CSF measures, Aβ levels were lower in the Aβ^+^ groups regardless of the stage along the AD continuum, whereas p-tau181 and t-tau levels increased with disease severity along the AD continuum (CU-Aβ^−^ < CU-Aβ^+^ < MCI-Aβ^+^ < ADD-Aβ^+^).

**Table 1:** Clinical characteristics of study participants

Characteristics	CU–Aβ^−^	CU–Aβ^+^	MCI–Aβ^+^	ADD–Aβ^+^	*P* value*
No. of participants	76	45	89	31	NA
No. of women (%)	39 (51)	23 (51)	47 (53)	22 (71)	0.220
Age at MRI scan ± SD, years	74.2 ± 6.2	74.4 ± 7.1	72.2 ± 7.3	73.1 ± 6.5	0.197
Educational attainment, years	16.4 ± 2.3	16.1 ± 2.1	16.5 ± 2.4	16.2 ± 2.3	0.751
No. of APOE ε4 allele (%)	7 (9)	15 (33)	38 (43)	20 (65)	0.003^a,b,c^
Neuropsychological assessments					
CDR, global score (Q1, Q3)	0 (0,0)	0 (0,0)	0.5 (0.5,0.5)	1.0 (1.0,1.0)	0.002^b,d,e,f^
CDR, sum of boxes (Q1, Q3)	0 (0,0)	0.5 (0,0.5)	1.0 (1.0,2.5)	4.0 (2.5,5.5)	< 0.001^b,c,d,e,f^
MMSE score ± SD	29.2 ± 1.0	28.6 ± 2.1	25.8 ± 4.5	21.6 ± 5.8	< 0.001^b,c,d,e,f^
MoCA score ± SD	27.6 ± 3.1	26.8 ± 3.4	22.1 ± 4.8	16.8 ± 6.3	< 0.001^b,c,d,e,f^
CSF measures					
Aβ1–42, pg/mL	1699 ± 559	792 ± 294	751 ± 271	688 ± 363	< 0.001^a,b,c^
p-tau181, pg/mL	19.7 ± 9.8	20.4 ± 8.8	23.5 ± 7.9	43.4 ± 14.1	< 0.001^c,e,f^
T-tau, pg/mL	211 ± 91	232 ± 83	256 ± 78	427 ± 162	< 0.001^c,e,f^

Aβ, amyloid β; ADD, Alzheimer’s disease dementia; APOE, apolipoprotein; CDR, clinical dementia rating; CSF, cerebrospinal fluid; CU, cognitively unimpaired; FDR, false discovery rate; MCI, mild cognitive impairment; MMSE, Mini-Mental State Examination; MoCA, Montreal Cognitive Assessment; Q1, Q3; quartile 1, quartile 3; SD, standard deviation.

*P values represent group comparisons based on ANOVA or Kruskal–Wallis tests.

^a^For the comparison between the CU-Aβ^−^ and CU-Aβ^+^ groups (FDR-corrected *P* < 0.05).

^b^For the comparison between the CU-Aβ^−^ and MCI-Aβ^+^ groups (FDR-corrected *P* < 0.05).

^c^For the comparison between the CU-Aβ^−^ and ADD-Aβ^+^ groups (FDR-corrected *P* < 0.05).

^d^For the comparison between the CU-Aβ^+^ and MCI-Aβ^+^ groups (FDR-corrected *P* < 0.05).

^e^For the comparison between the CU-Aβ^+^ and ADD-Aβ^+^ groups (FDR-corrected *P* < 0.05).

^f^For the comparison between the MCI-Aβ^+^ and ADD-Aβ^+^ groups (FDR-corrected *P* < 0.05).

### First-order radiomic features

Across the 241 participants, first-order radiomic features derived from their HighResHippo MRIs demonstrated considerable variability in each hippocampal subfield, with several measures showing systematic differences along the AD continuum. Notably, these patterns were particularly pronounced in the subiculum (Supplementary Fig. 4). The standardized mean signal intensity in the subiculum across all participants was 0.49 ± 0.21 (median, 0.47; range, –0.12 to 1.04), with the highest mean observed in ADD-Aβ^+^ (0.59 ± 0.20) and the lowest in CU-Aβ^−^ (0.41 ± 0.19). Meanwhile, measures of dispersion, such as the interquartile range, were largest in MCI-Aβ^+^ (0.30 ± 0.05) and smallest in ADD-Aβ^+^ (0.25 ± 0.03), indicating greater heterogeneity in advanced disease stages. The mean first-order entropy values (Supplementary Fig. 5) were highest in CU-Aβ^−^ (0.32 ± 0.21) and lowest in ADD-Aβ^+^ (0.24 ± 0.18), consistent with a loss of fine-scale heterogeneity as disease progresses, although this difference did not reach statistical significance. Higher-order moments—skewness and kurtosis—also varied across the AD continuum. Kurtosis was higher in Aβ^+^ groups than in CU-Aβ^−^ (3.42 ± 0.72), with the highest mean observed in CU-Aβ^+^ (3.88 ± 1.54). Skewness increased towards ADD-Aβ+ (0.15 ± 0.42), indicating progressively more peaked and slightly right-tailed intensity distributions in later stages (Supplementary Fig. 6).

### Volumetric and thickness measures

**Figure 2:**
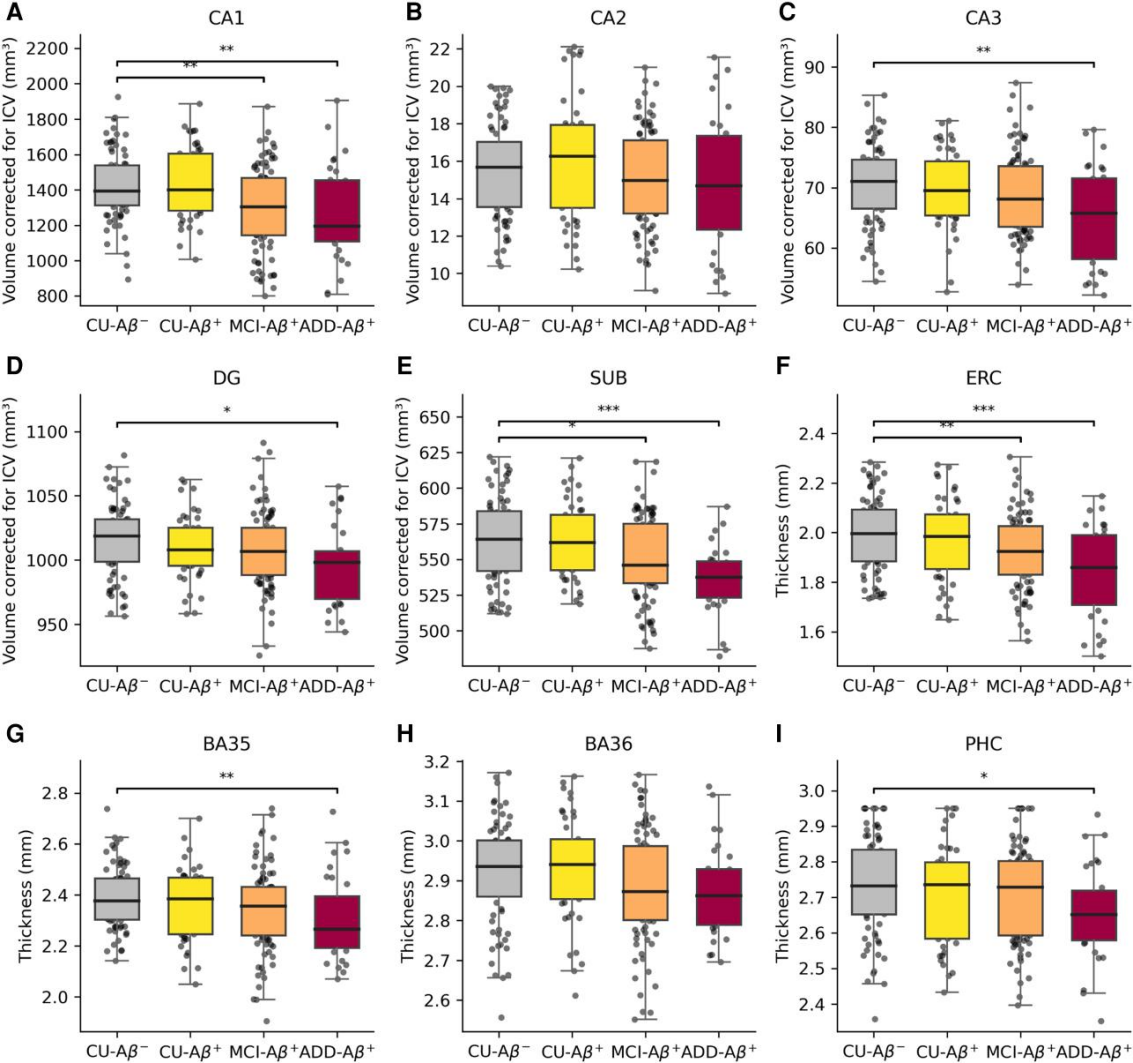
**Group-wise comparisons of volumes and cortical thickness.** The volumes of hippocampal subfields (CA1 [A], CA2 [B], CA3 [C], DG [D], SUB [E]) and median thickness of MTL cortical regions (ERC [F], BA35 [G], BA36 [H], PHC [I]) are plotted for the CU-Aβ^−^, CU-Aβ^+^, MCI-Aβ^+^, and ADD-Aβ^+^ groups. Hippocampal subfield volumes are standardized by intracranial volume. Group-wise comparisons were conducted using one-way analysis of variance with post hoc pairwise comparisons corrected for multiple testing using the Benjamini–Hochberg FDR procedure. The sample sizes were CU-Aβ^−^ (*N* = 76), CU-Aβ^+^ (*N* = 45), MCI-Aβ^+^ (*N* = 89) and ADD-Aβ^+^ (*N* = 31). Each data point represents the volume or cortical thickness value from a single participant. Significant differences after correction for multiple comparisons using FDR are denoted as *FDR-corrected *P* value < 0.05, **FDR-corrected *P* value < 0.01 and ***FDR-corrected *P* value < 0.001. ADD, Alzheimer’s disease dementia; BA, Brodmann area; CA, cornu ammonis; DG, dentate gyrus; ERC, entorhinal cortex; FDR, false discovery rate; ICV, intracranial volume; MTL, medial temporal lobe; PHC, parahippocampal cortex; SUB, subiculum.

Volumetric and cortical thickness measures of hippocampal subfields revealed atrophic changes along the AD continuum (Fig. 2). The volumes of hippocampal subfields, except for CA2, were progressively reduced from CU-Aβ^−^ to ADD-Aβ^+^. In the subiculum, a significant decline was observed between CU-Aβ^−^ (564.2 ± 29.5 mm^3^) and ADD-Aβ^+^ (536.6 ± 23.8 mm^3^), representing an approximately 4.9% reduction (FDR-corrected *P* value < 0.001). Similar trends were observed for cortical thickness, with ERC showing a significant difference between CU-Aβ^−^ (2.00 mm; 95% CI 1.88 to 2.09 mm) and ADD-Aβ^+^ (1.86 mm; 95% CI 1.71 to 1.99), representing an approximately 7.0% reduction (FDR-corrected *P* value < 0.001).

### Texture-based radiomic features

**Figure 3:**
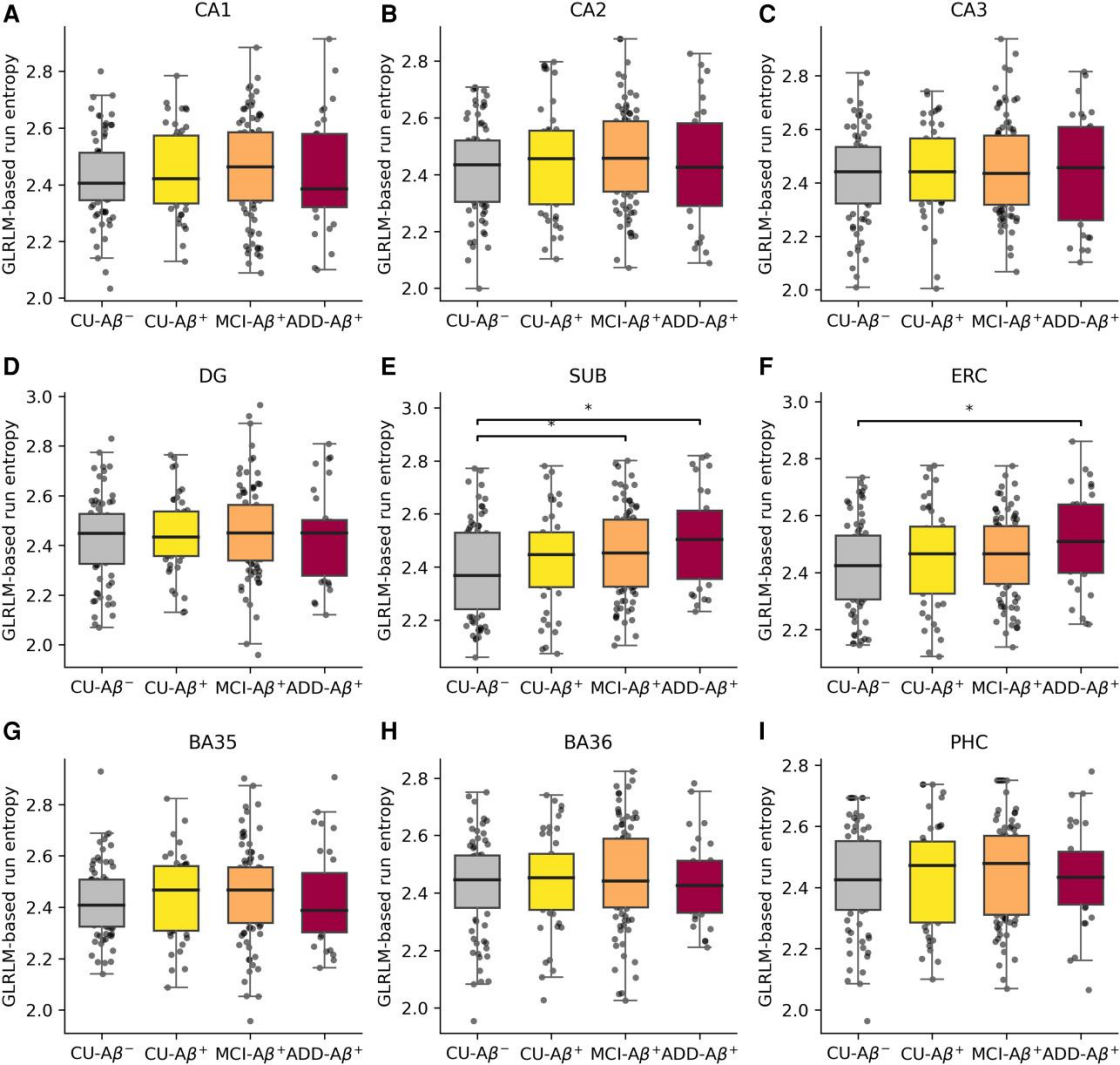
**Group-wise comparisons of GLRLM-based run entropy.** The values of hippocampal subfields (CA1 [A], CA2 [B], CA3 [C], DG [D], SUB [E]) and MTL cortical regions (ERC [F], BA35 [G], BA36 [H], PHC [I]) are plotted for the CU-Aβ^−^, CU-Aβ^+^, MCI-Aβ^+^ and ADD-Aβ^+^ groups. Group-wise comparisons were conducted using one-way analysis of variance with post hoc pairwise comparisons corrected for multiple testing using the Benjamini–Hochberg FDR procedure. The sample sizes were CU-Aβ^−^ (*N* = 76), CU-Aβ^+^ (*N* = 45), MCI-Aβ^+^ (*N* = 89) and ADD-Aβ^+^ (*N* = 31). Each data point represents the GLRLM-based run entropy from a single participant. Group differences were observed in SUB and ERC, where ADD-Aβ^+^ individuals exhibited higher run entropy compared to CU-Aβ^−^. Significant differences after correction for multiple comparisons using FDR are denoted as *FDR-corrected *P* value < 0.05. ADD, Alzheimer’s disease dementia; BA, Brodmann area; CA, cornu ammonis; DG, dentate gyrus; ERC, entorhinal cortex; FDR, false discovery rate; GLRLM, grey-level run-length matrix; MTL, medial temporal lobe; PHC, parahippocampal cortex; SUB, subiculum.

Texture-based radiomic features from hippocampal subfields showed marked variability across the AD continuum. GLCM-derived contrast values in the subiculum were lowest in ADD-Aβ^+^ (0.06 ±0.10) compared with CU-Aβ^+^ (0.09 ± 0.20), suggesting reduced local intensity variation in advanced stages. GLRLM-derived features further characterized run-length properties. Specifically, GLRLM-based run entropy demonstrated a broad spread across groups in the subiculum (mean range: 2.39–2.51), with the lowest value in CU-Aβ^−^ (2.39 ± 0.18) and the highest value in ADD-Aβ^+^ (2.51 ± 0.18; Fig. 3). Here, lower run entropy reflects more uniform and long-run-dominated textures, whereas higher run entropy indicates more heterogeneous and irregular run-length (Supplementary Fig. 7).

**Figure 4:**
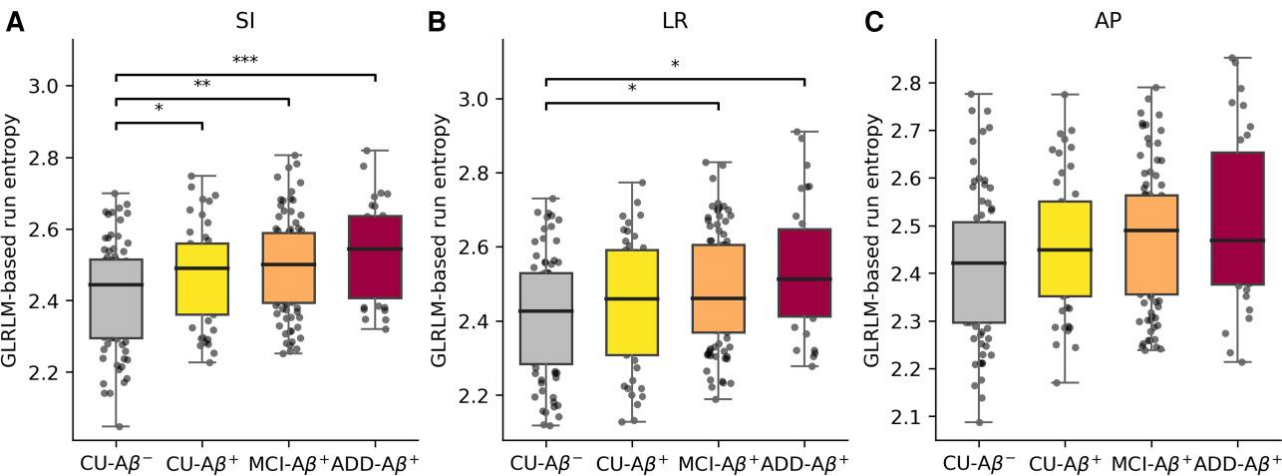
**Group-wise comparisons of GLRLM-based run entropy along three orientations.** The three orientations are defined as follows: SI corresponds to the superior–inferior direction (**A**), LR to the left–right direction (**B**) and AP to the anterior–posterior direction (**C**) within the subiculum. Group-wise comparisons were conducted using one-way analysis of variance with post hoc pairwise comparisons corrected for multiple testing using the Benjamini–Hochberg FDR procedure. The sample sizes were CU-Aβ^−^ (*N* = 76), CU-Aβ^+^ (*N* = 45), MCI-Aβ^+^ (*N* = 89) and ADD-Aβ^+^ (*N* = 31). Each data point represents the GLRLM-based run entropy along three orientations within the subiculum from a single participant. Compared with CU–Aβ^−^, GLRLM-based run entropy values in the SI component showed increases of +0.12 in CU–Aβ^+^, +0.25 in MCI–Aβ^+^, and +0.36 in ADD–Aβ^+^. In the LR component, MCI–Aβ^+^ showed a + 0.03 increase and ADD–Aβ^+^ a + 0.09 increase compared with CU–Aβ^−^. In contrast, the AP component did not show notable group differences. Significant differences after correction for multiple comparisons using FDR are denoted as *FDR-corrected *P* value < 0.05, **FDR-corrected *P* value < 0.01, and ***FDR-corrected *P* value < 0.001. ADD, Alzheimer’s disease dementia; FDR, false discovery rate; GLRLM, grey-level run-length matrix.

When GLRLM-based run entropy was decomposed into orientation-specific components, significant group differences were most pronounced in the SI component of the subiculum, indicating strong sensitivity to vertical directional properties (Fig. 4). Compared with CU–Aβ^−^, GLRLM-based run entropy values in the SI component showed increases of +0.12 in CU–Aβ^+^, +0.25 in MCI–Aβ^+^, and +0.36 in ADD–Aβ^+^. In the LR component, MCI–Aβ^+^ showed a + 0.03 increase and ADD–Aβ^+^ a + 0.09 increase, compared with CU–Aβ^−^. By contrast, these values in the AP component did not show notable group effects.

### Associations between texture features and cognition

**Figure 5:**
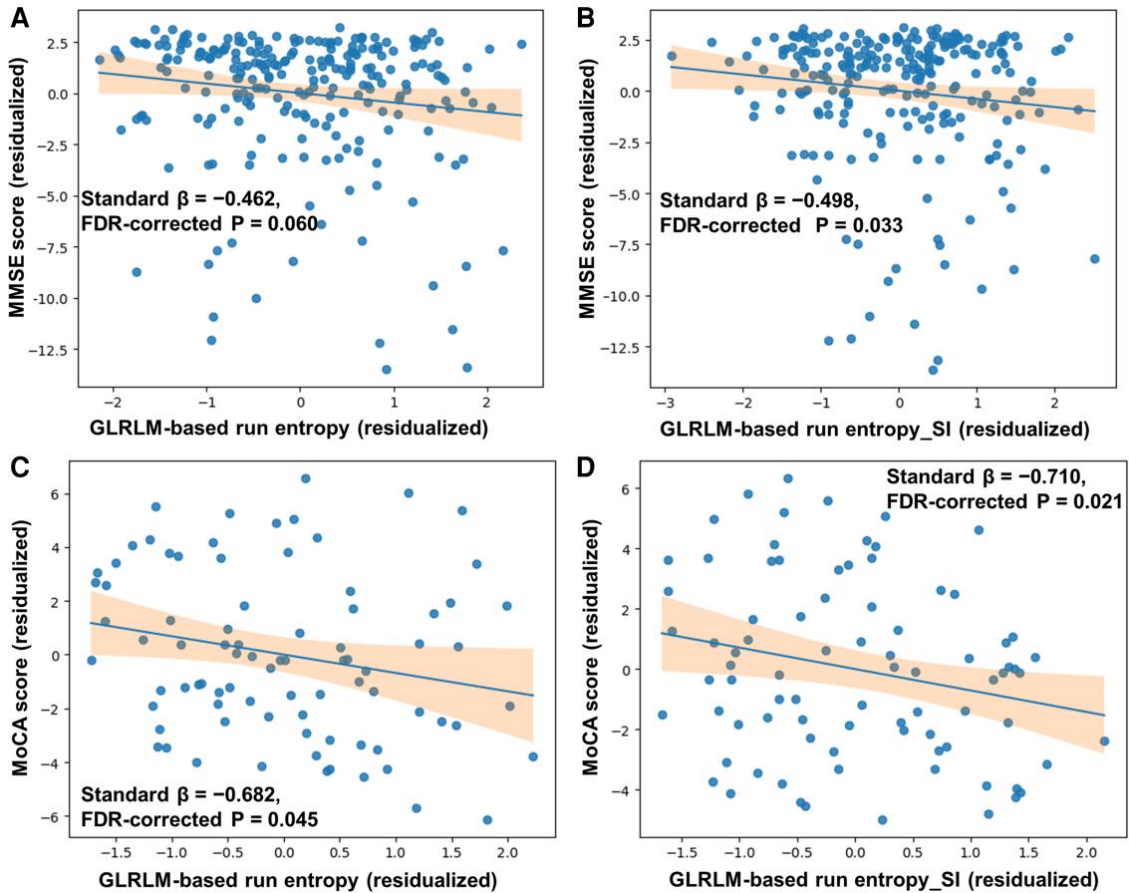
**Associations between texture features and cognition.** Associations between cognitive scores and texture features were evaluated using multiple linear regression models adjusted for age, sex, years of education and APOE ε4 carrier status. The sample sizes for the regression analyses were *N* = 238 for the MMSE and *N* = 83 for the MoCA, reflecting the availability of cognitive data. Each data point represents an individual participant, plotted using covariate-adjusted (residualized) values of both cognitive scores and imaging features. (**A**) Residualized MMSE scores exhibited an inverse trend, with negative coefficients for residualized GLRLM-based run entropy values in the subiculum (Standard β = −0.462; 95% CI −0.942 to 0.019; FDR-corrected *P* = 0.060). (**B**) The association reached statistical significance when analyses were restricted to the SI direction (Standard β = −0.498; 95% CI −1.013 to −0.025; FDR-corrected *P* = 0.033). (**C, D**) Residualized MoCA scores demonstrated significant inverse associations with residualized GLRLM-based run entropy values in the subiculum in both the overall (Standard β = −0.682; 95% CI −1.349 to −0.014; FDR-corrected *P* = 0.045) and the SI-restricted analysis (Standard β = −0.710; 95% CI −1.416 to −0.038; FDR-corrected *P* = 0.021). FDR, false discovery rate; GLRLM, grey-level run-length matrix. MMSE, Mini-Mental State Examination; MoCA, Montreal Cognitive Assessment; SI, superior–inferior.

Texture-based metrics derived from hippocampal subfields demonstrated significant associations with cognitive performance, with patterns that aligned with group-level differences along the AD continuum. Residualized MMSE scores exhibited an inverse trend, with negative coefficients for residualized GLRLM-based run entropy values in the subiculum (standard β = −0.462; 95% CI −0.942 to 0.019; FDR-corrected *P* = 0.060; Fig. 5A). This association reached statistical significance when analyses were restricted to the SI direction (standard β = −0.498; 95% CI −1.013 to −0.025; FDR-corrected *P* = 0.033; Fig. 5B). In contrast, residualized MoCA scores demonstrated significant inverse associations with residualized GLRLM-based run entropy values in the subiculum in both the overall (standard β = −0.682; 95% CI −1.349 to −0.014; FDR-corrected *P* = 0.045; Fig. 5C) and the SI-restricted analysis (standard β = −0.710; 95% CI −1.416 to −0.038; FDR-corrected *P* = 0.021; Fig. 5D). Residuals were visualized to assess the validity of model assumptions (Supplementary Fig. 8).

### Associations between texture features and CSF tau levels

Similar to the findings for cognition, residualized CSF tau levels were significantly associated with residualized GLRLM-based run entropy values (p-tau181: standard β = 1.341; 95% CI 0.103 to 2.580; FDR-corrected *P* = 0.034; Supplementary Fig. 9A, t-tau: standard β = 13.934; 95% CI 2.425 to 25.443; FDR-corrected *P* = 0.018; Supplementary Fig. 9B), as well as those restricted to the SI direction (p-tau181: standard β = 2.307; 95% CI 0.767 to 3.848; FDR-corrected *P* = 0.003; Supplementary Fig. 9C, t-tau: standard β = 18.822; 95% CI 4.698 to 32.947; FDR-corrected *P* = 0.009; Supplementary Fig. 9D). Residuals were visualized to assess the validity of model assumptions (Supplementary Fig. 10).

## Discussion

In the retrospective, multicentre, cross-sectional study using the ADNI database, texture-based radiomic features derived from *in vivo* high-resolution T2-weighted ‘HighResHippo’ MRI captured microstructural alterations of hippocampal subregions that tracked the AD continuum and related to cognition. Across 241 ADNI participants stratified by CSF Aβ status and clinical diagnosis, first-order statistical distributions and morphometrics (i.e. volume and thickness) revealed expected vulnerability of the subiculum and ERC, providing anatomical context for texture findings. Critically, texture metrics—especially GLRLM-based run entropy in the subiculum—demonstrated progressive, orientation-specific differences across groups, and inverse associations with cognitive performance after covariate adjustment for age, sex, years of education and APOE ε4 carrier status. The SI-direction component of run entropy was most informative, showing significant negative associations with both MMSE and MoCA. Our findings support the potential utility of hippocampal subfield radiomics as imaging correlates of microstructural disorganization along the AD continuum, with the subiculum emerging as a key locus where texture alterations are associated with cognitive performance.^26^

The distributional properties of subicular intensity measures demonstrated distinct alterations across the AD continuum. In the CU-Aβ^−^ group, both skewness and kurtosis approximated normal distributions, indicating relative homogeneity of tissue characteristics. By contrast, Aβ positivity and subsequent disease stages were associated with greater asymmetry in skewness and increasingly heavy-tailed kurtosis distributions, particularly evident in the MCI-Aβ^+^ and ADD-Aβ^+^ groups. These findings suggest that subicular signal intensity becomes more heterogeneous with disease severity, possibly reflecting underlying pathophysiological processes such as neuronal degeneration, gliosis or Aβ-related changes.^27,28^ Collectively, these findings suggest that first-order intensity characteristics not only vary between individuals but also follow systematic trends along the AD continuum, with the subiculum showing signal changes that may capture microstructural alterations preceding overt atrophy.

In this study, the first-order entropy and GLRLM-based run entropy demonstrated opposite trends, which likely reflects that these metrics capture different aspects of texture characteristics. While first-order entropy reflects the randomness of voxel intensity distribution based on the histogram,^29^ run entropy describes the complexity of spatial arrangement and continuity of intensities.^30^ Thus, with disease progression, the intensity distribution may become more homogeneous (reduced first-order entropy), whereas the underlying microstructural organization becomes more disrupted and irregular (increased run entropy), highlighting a dual aspect of pathological changes.^31^ Importantly, GLRLM-based run entropy is a non-specific texture metric that quantifies spatial irregularity of signal intensity and should not be interpreted as a direct measure of perforant pathway degeneration. Rather, it likely reflects a composite of microstructural alterations, including neuronal loss, gliosis, microvascular changes and age-related tissue reorganization.^32^

An important strength of the study was the addition of orientation-specific analysis of the GLRLM-based run entropy in the subiculum, whereby these values were decomposed into SI, LR and AP components. This approach was motivated by the underlying anatomical organization of entorhinal-hippocampal fibre tracts, with the perforant path fibres primarily oriented in the SI direction.^16,17^ Our results demonstrated that the SI component was particularly sensitive to AD-related changes, showing progressive disruption of run-length homogeneity along the AD continuum, whereas the AP component did not reveal significant group differences. The lack of robust findings in the AP direction is plausibly explained by the lower through-slice resolution of 2 mm, which may have limited the ability to capture subtle structural heterogeneity. These findings highlight that directional decomposition of texture features can provide biologically meaningful insights into hippocampal circuit alterations related to the vulnerability of perforant pathway-related microstructures during the early stages of AD.^12,13^

When interpreting the directional changes within the subiculum, it is also important to consider the potential contribution of the capillary vasculature.^33^ For instance, capillary rarefaction and pericyte loss have been reported in the hippocampal formation even before overt atrophy or amyloid deposition becomes apparent.^34,35^ Such early vascular dysregulation can lead to chronic hypoperfusion, tissue hypoxia and gliosis, resulting in subtle disorganization of the neuropil. Because the capillary networks in the subiculum largely follow the SI axis, these microcirculatory changes could preferentially alter the SI component of run entropy.

Our study has several limitations. First, its retrospective, cross-sectional design using ADNI precludes causal inference and prevents assessment of within-person change; future longitudinal work with repeated HighResHippo scans should be followed. Within the broader context of recent tau imaging and plasma biomarker studies, hippocampal texture alterations may evolve in parallel with, or partially independently from, regional tau accumulation.^36,37^ Determining whether these processes track together or diverge temporally will require future longitudinal and multimodal investigations. Second, the lack of a formal test–retest assessment should be noted. Test–retest MRI data were not consistently available in the present cohort, preventing evaluation of repeatability for texture-based features. The segmentation relied on ASHS with dual-contrast inputs and visual quality control, but residual label errors in small hippocampal cortices/subfields are possible and could bias texture estimates. Future studies using ADNI test–retest datasets or independent cohorts will be required to address this important methodological aspect. Third, cognition was indexed by MMSE and MoCA; more granular domain scores (e.g. episodic memory composites) could sharpen structure–function links. Fourth, unmeasured confounders, such as white matter hyperintensity due to vascular burden, medications and comorbid neurodegeneration, were not explicitly modelled and may contribute to the observed associations. Finally, the unilateral focus on the left hippocampus represents an important limitation. Hemispheric asymmetry in hippocampal vulnerability may be influenced not only by AD pathology but also by coexisting age-related tauopathies, such as argyrophilic grain disease, which can exhibit hemispheric predominance.^38^

In conclusion, texture-based radiomic analyses of hippocampal subfields using *in vivo* HighResHippo T2-weighted MRI revealed microstructural alterations associated with disease severity and cognition that were not detectable by volumetric measures alone. GLRLM-based run entropy in the subiculum—particularly its SI-oriented component, consistent with perforant pathway anatomy—exhibited progressive differences along the AD continuum and showed inverse associations with the cognitive function scores. Future longitudinal and externally validated studies are warranted to establish the clinical utility of this approach.

## Supplementary Material

fcag164_Supplementary_Data

## Data Availability

Anonymized data used in the analyses presented in this report are available upon request from qualified investigators (adni.loni.usc.edu). All Python code used for statistical analyses in this study is provided as Supplementary material (Supplementary_Code.txt).
