# Supplementary material for: Hippocampal subregional texture features associated with Alzheimer’s disease severity and cognition
Source: Brain Commun. 2026 May 6;8(3):fcag164. doi: 10.1093/braincomms/fcag164 (PMC13184691; doi:10.1093/braincomms/fcag164)
Supplement: fcag164_Supplementary_Data [file fcag164_supplementary_data.zip › Supplementary_material.pdf]

Supplemental Materials

Appendix S1

CSF biomarkers

CSF amyloid-β (Aβ) biomarkers were used to classify participants based on their Aβ pathology for subsequent cross-sectional analyses. Specifically, levels of $A\beta_{1\text{–}42}$ in the CSF were used as a grouping variable to define their Aβ status. Participants were categorized as Aβ-positive ($A\beta^{+}$) if their CSF $A\beta_{1\text{–}42}$ concentration was less than or equal to 980 pg/mL, based on established cutoffs provided by the ADNI biomarker core.[1] This threshold was derived using the Roche Elecsys immunoassay platform, which employs an electrochemiluminescence-based detection method offering high sensitivity and reproducibility across multiple study sites.[2] Meanwhile, CSF tau biomarkers, including phosphorylated tau at threonine 181 (p-tau181) and total tau (t-tau), were examined as indicators of tau pathology and neurodegeneration. These markers were used to exclude individuals with non-Alzheimer's pathologic changes (i.e., CSF Aβ-negative and at least one of CSF p-tau181- or t-tau-positive) from the present study. By excluding these cases, this study was able to focus more specifically on participants along the AD continuum.[3] CSF tau levels were also used to characterize disease severity across each diagnostic group.

Appendix S2

HighResHippo MRI protocol

The HighResHippo MRI protocol is a high-resolution T2-weighted imaging sequence specifically designed to visualize the internal architecture of the hippocampus and its

subfields with exceptional anatomical details.[4] It typically employs a 2D turbo spin echo sequence with an in-plane resolution of approximately 0.4 × 0.4 mm and a slice thickness of 2 mm, acquired perpendicular to the long axis of the hippocampus (Supplementary Fig. 1A). The detailed imaging protocols are provided at the ADNI website (http://adni.loni.usc.edu/ methods/ documents/). The HighResHippo sequence has been widely implemented in multi-center studies as ADNI, which is expected to be critical for studies investigating early neurodegenerative changes in AD and related dementias.[5] The high spatial resolution and consistent orientation across individuals would make it ideal for the clear delineation of hippocampal subregions and extraction of these radiomic features.[6]

Appendix S3

Hippocampal segmentation

We performed hippocampal subfield segmentation using the Automatic Segmentation of Hippocampal Subfields (ASHS) software (version 2.0.0), which provides accurate and robust parcellation of hippocampal anatomy through a multi-atlas label fusion approach.[7] High-resolution T2-weighted coronal images acquired using the standardized HighResHippo protocol were utilized as the primary input, along with co-registered T1-weighted images. Segmentation was conducted using the ASHS-PMC-T1T2 atlas (https://www.nitrc.org/projects/ashs), which is optimized for older adults and individuals with cognitive impairment and supports dual-modality input. The segmentation yields detailed delineation of hippocampal and medial temporal lobe (MTL) subregions, including CA1, CA2, CA3, dentate gyrus, subiculum (comprising subiculum proper, presubiculum, and parasubiculum), ERC, and parahippocampal cortex (PHC), subdivided into Brodmann areas (BA) 35 and 36. All processing was conducted in the subject's native anatomical space to preserve spatial precision.[8]

Following automated segmentation, all outputs were visually inspected for quality assurance (Y.U., with 14 years of brain MRI research). The quality control process involved overlaying the segmentation masks onto the original T2-weighted images to assess anatomical accuracy and to identify mislabeling, misalignment, or significant artifacts. Segmentations showing gross misregistration, missing labels, or substantial motion artifacts that compromised anatomical boundaries were excluded from further analysis. This quality control step ensured the reliability of region-specific radiomic feature extraction analyses used in subsequent statistical modeling.[9] Representative examples are shown in Supplementary Fig. 10. Following quality control procedures, 18 cases with incomplete segmentation and 9 cases with motion artifacts were excluded from further analyses.

Appendix S4

Radiomic feature extraction

Image preprocessing steps were applied to reduce inter-subject variability and acquisition-related artifacts in the HighResHippo MRI. Specifically, N4 bias field correction (ANTs implementation) was first applied to minimize low-frequency intensity non-uniformity. Subsequently, voxel intensity normalization was performed by z-score standardizing each subject's T2-weighted signal intensity based on the whole-brain intensity distribution.[9] After these preprocessing steps, radiomic features were computed on MTL regions segmented with the ASHS algorithm (Supplementary Fig. 1B). For our hypothesis-driven radiomic analysis, PyRadiomics (version 3.0.1), an open-source Python package compliant with the Image Biomarker Standardization Initiative, was used. Feature categories included first-order intensity statistics (e.g., mean, dispersion, entropy, skewness, and kurtosis), shape descriptors (e.g., volume and thickness), and texture features derived from the gray-level run-length matrix (GLRLM), which characterizes the distribution of consecutive voxels with

identical intensities, thereby capturing run-length patterns of uniform signal.[10] ROI volumes were normalized to intracranial volume (ICV) to account for individual differences in head size, with ICV obtained from whole-brain segmentation of the T1-weighted images. For texture computation, each ROI was resampled to isotropic voxel spacing (0.4 mm^3) to ensure consistency, and intensities were discretized into 32 gray levels using a fixed bin-width approach.[11] Then, the GLRLM-based run entropy was analyzed in an orientation-dependent manner by decomposing it into superior–inferior (SI), left–right (LR), and anterior–posterior (AP) components. This approach enabled the measure to more directly reflect directional properties of underlying neural fibers within the hippocampal subfields. All feature extraction was conducted in 3D using the original spatial orientation of each subject's native image. In addition to PyRadiomics features, cortical thickness was computed for MTL cortical regions (e.g., ERC, BA35, BA36, and PHC) using the cortical reconstruction for ASHS,[12] a software pipeline that applies surface-based cortical modeling and registration techniques. All Python code used for statistical analyses in this study is provided as Supplementary_Code.txt.

Appendix S5

Image Biomarker Standardization Initiative (IBSI)-style radiomics reporting parameters

A. Image acquisition/inputs

Category	Item	Value/Setting
Imaging	Modality/sequence	ADNI "HighResHippo" coronal T2-weighted turbo spin echo (2D)
Imaging	Native voxel spacing (mm)	0.4 × 0.4 × 2.0
Imaging	Slice gap	None (contiguous slices)
Imaging	Reconstruction/filtering	None reported
Inputs	Images used for segmentation	Dual-contrast ASHS (co-registered T1- and T2-weighted images)
Inputs	Images used for radiomics	Preprocessed HighResHippo T2-weighted

Category	Item	Value/Setting
		images

B. Segmentation/ROI definition

Category	Item	Value/Setting
ROI	Segmentation method	Automatic Segmentation of Hippocampal Subfields (ASHS) v2.0.0
ROI	Atlas	PMC-T1T2 atlas
ROI	ROIs analyzed	CA1–CA3, dentate gyrus, subiculum, ERC, BA35, BA36, PHC
ROI	ROI space	Resampled isotropic T2 space
ROI	ROI label handling	Binary masks; no partial volume modeling
ROI QC	QC method	Visual inspection by an expert reader
ROI QC	Exclusion criteria	Incomplete segmentation; substantial motion artifacts
ROI QC	QC reporting	Overall and region-specific exclusion rates

C. Preprocessing (intensity/bias correction)

Category	Item	Value/Setting
Preprocessing	Bias-field correction	N4 bias correction
Preprocessing	Intensity normalization	Z-score normalization (per image)
Preprocessing	Intensity scaling/clamping	None
Preprocessing	Denoising	None
Preprocessing	Registration	T1–T2 rigid registration performed within the ASHS pipeline

D. Resampling/interpolation

Category	Item	Value/Setting
Resampling	Target voxel spacing (mm)	0.4 × 0.4 × 0.4 (isotropic)
Resampling	Resampling grid	Isotropic reference grid
Interpolation	Image interpolation method	B-spline interpolation
Interpolation	Interpolation kernel/order	Cubic B-spline (order = 3)
Interpolation	Mask interpolation	Nearest-neighbor
Notes	Anisotropy handling	3D texture features computed after isotropic resampling

F. Feature extraction

Category	Item	Value/Setting
Feature set	Classes extracted	First-order, shape, texture (GLCM, GLRLM)
Texture	GLRLM computation	3D GLRLM, voxel distance = 1
Texture	GLRLM directions	13 3D directions
Texture	Directional decomposition	SI, LR, AP components analyzed separately

Category	Item	Value/Setting
		in subiculum
Aggregation	Direction aggregation	Direction-specific features reported (no averaging)
Implementation	Software	PyRadiomics v3.0.1
Implementation	Feature definitions	IBSI-compliant via PyRadiomics
Reproducibility	Random seed	Not applicable (deterministic pipeline)

G. Harmonization/statistics

Category	Item	Value/Setting
Harmonization	Method	ComBat (empirical Bayes)
Harmonization	Batch variable	Scanner vendor (Siemens, GE, Philips)
Harmonization	Biological covariates preserved	Age, sex, years of education, APOE ε4 carrier status
Diagnostics	Pre/post evaluation	Vendor-stratified distributions and variance

Supplementary Figures

Supplementary Fig. 1. Hippocampal subfield segmentation and ROI extraction for texture analysis. (A) A high-resolution coronal T2-weighted FSE sequence was scanned with an in-plane resolution of approximately 0.4 × 0.4 mm and a slice thickness of 2 mm, acquired perpendicular to the long axis of the hippocampus. The white dashed box surrounds the left side of MTL. (B) Magnified coronal view of the hippocampal subfield segmentation: CA1 (orange), CA3 (light pink), DG (green), SUB (red), ERC (cyan), and BA35 (magenta). (C) GLRLM based on run entropy decomposed into SI, LR, and AP orientations. AP, anterior–posterior; BA, Brodmann area; CA, cornu ammonis; DG, dentate gyrus; FSE, fast spin echo; GLRLM, gray level run length matrix; LR, left–right; MTL, medial temporal lobe; R, right; ROI, region of interest; SI, superior–inferior; SUB, subiculum.

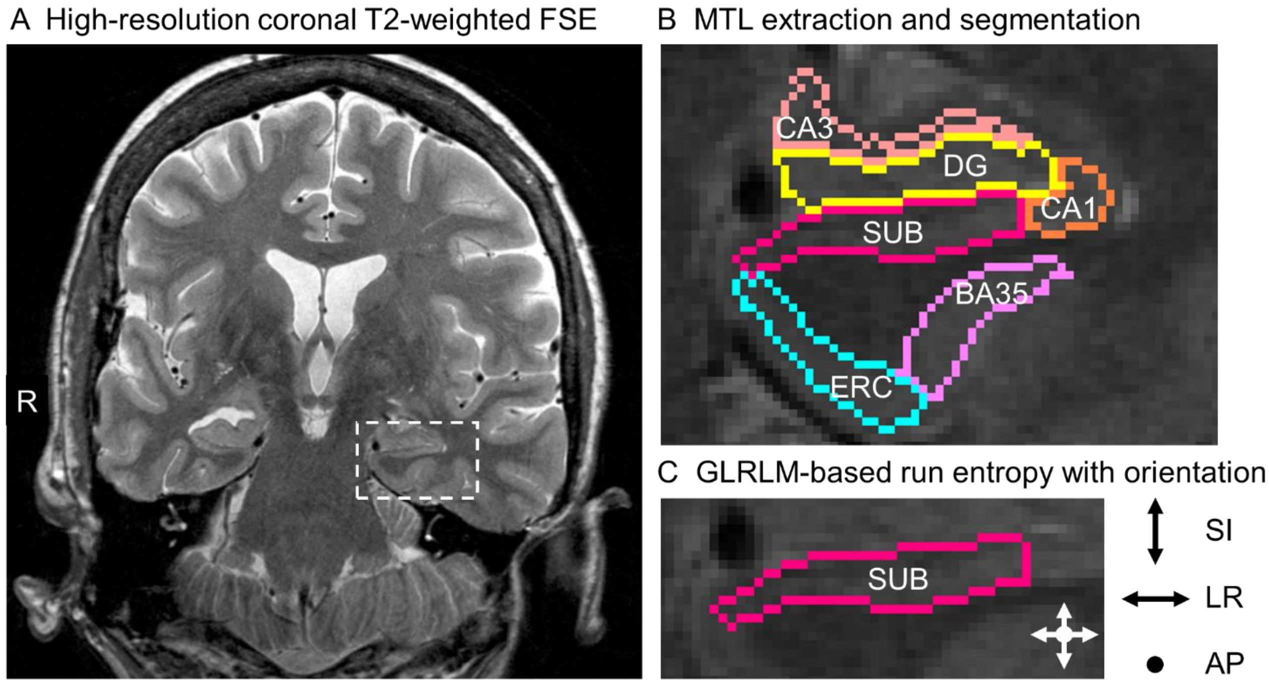

Supplementary Fig. 2. Following automated segmentation, the quality control process involved overlaying the segmentation masks onto the original T2-weighted images to assess anatomical accuracy and to identify mislabeling, misalignment, or significant artifacts. Segmentations showing gross misregistration, missing labels, or substantial motion artifacts that compromised anatomical boundaries were excluded from further analysis. Representative examples of (A) accepted segmentation, (B) incomplete segmentation, and (C) motion artifacts are shown. Following quality control procedures, 18 cases with incomplete segmentation and 9 cases with motion artifacts were excluded from further analyses.

(A) Accepted segmentation (B) Incomplete segmentation (C) Motion Artifacts

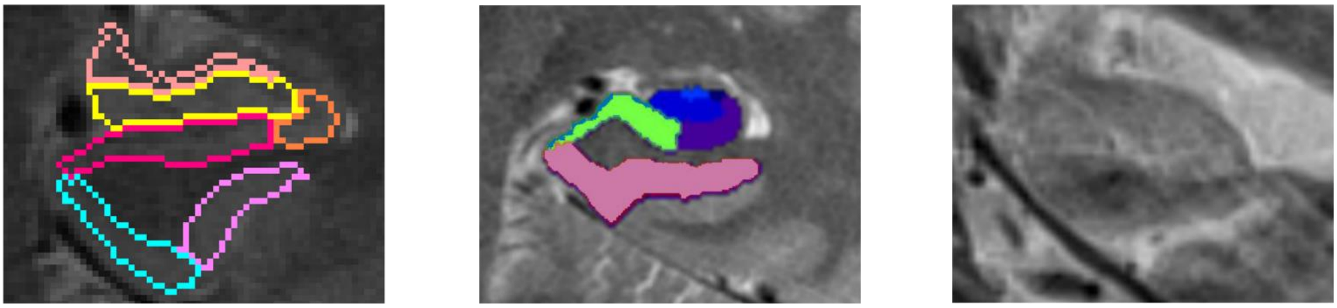

Supplementary Fig. 3. Effect of ComBat harmonization on scanner-related variability. (A) Pre-harmonization distributions of a representative radiomic feature, subicular gray-level run-length matrix (GLRLM)-based run entropy, stratified by scanner vendor (Siemens, GE, Philips). (B) Post-harmonization distributions following ComBat adjustment using scanner vendor as the batch variable. Violin plots display the distribution of individual participant values, with horizontal lines indicating the median and interquartile range. The sample sizes were: Siemens (n = 106), GE (n = 83), and Philips (n = 52). Each data point represents an individual participant (n = individual participants). (C) Variance decomposition analysis quantifying the proportion of feature variance attributable to scanner vendor before and after harmonization. The percentage of variance explained (η^2) was estimated using analysis of variance, demonstrating a reduction in vendor-related variance from 5.0% before ComBat to 0.0% after ComBat.

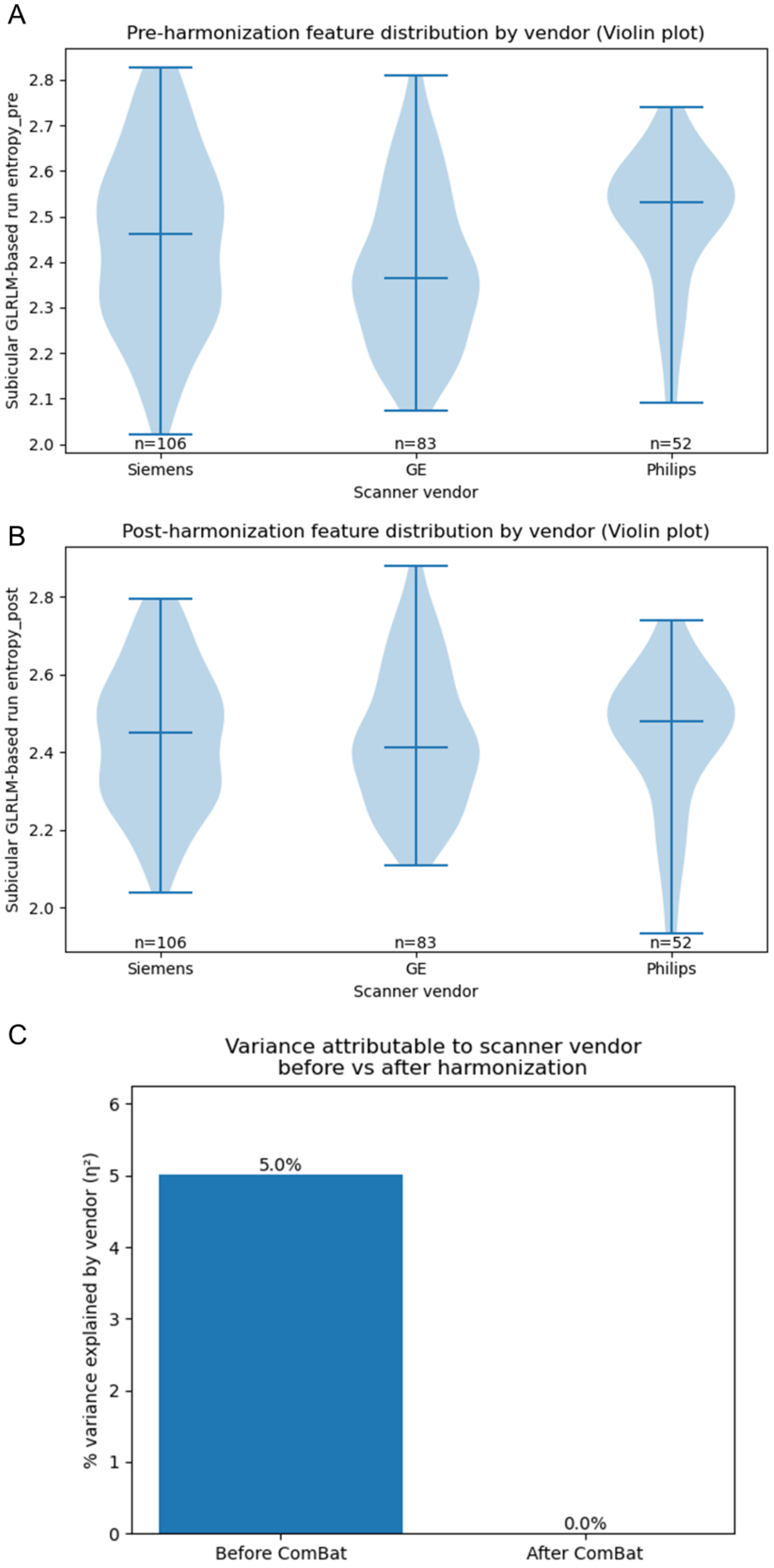
A
Pre-harmonization feature distribution by vendor (Violin plot)
Subicular GLRLM-based run entropy_pre
2.0
2.1
2.2
2.3
2.4
2.5
2.6
2.7
2.8
n=106
n=83
n=52
Siemens
GE
Philips
Scanner vendor
B
Post-harmonization feature distribution by vendor (Violin plot)
Subicular GLRLM-based run entropy_post
2.0
2.2
2.4
2.6
2.8
n=106
n=83
n=52
Siemens
GE
Philips
Scanner vendor
C
Variance attributable to scanner vendor
before vs after harmonization
% variance explained by vendor (η²)
0
1
2
3
4
5
6
5.0%
0.0%
Before ComBat
After ComBat

Supplementary Fig. 4. Group-wise distributions of standardized mean signal intensity in hippocampal subfields and medial temporal cortical regions. Z-scored mean signal intensity (standardized within subject using the whole-brain intensity distribution) was calculated for each ROI—CA1 (A), CA2 (B), CA3 (C), DG (D), SUB (E), ERC (F), BA35 (G), BA36 (H), and PHC (I)—from the HighResHippo T2-weighted MRI. Group-wise comparisons were conducted using one-way analysis of variance with post hoc pairwise comparisons corrected for multiple testing using the Benjamini–Hochberg FDR procedure. The sample sizes were CU-Aβ^- (N = 76), CU-Aβ^+ (N = 45), MCI-Aβ^+ (N = 89), and ADD-Aβ^+ (N = 31). Each data point represents the signal intensity from a single participant. SUB and ERC show the upward shifts along the AD continuum. *FDR-corrected P value < 0.05, **FDR-corrected P value < 0.01, and ***FDR-corrected P value < 0.001. AD, Alzheimer's disease; ADD, Alzheimer's disease dementia; BA, Brodmann area; CA, cornu ammonis; DG, dentate gyrus; ERC, entorhinal cortex; FDR, false discovery rate; MTL, medial temporal lobe; PHC, parahippocampal cortex; SUB, subiculum.

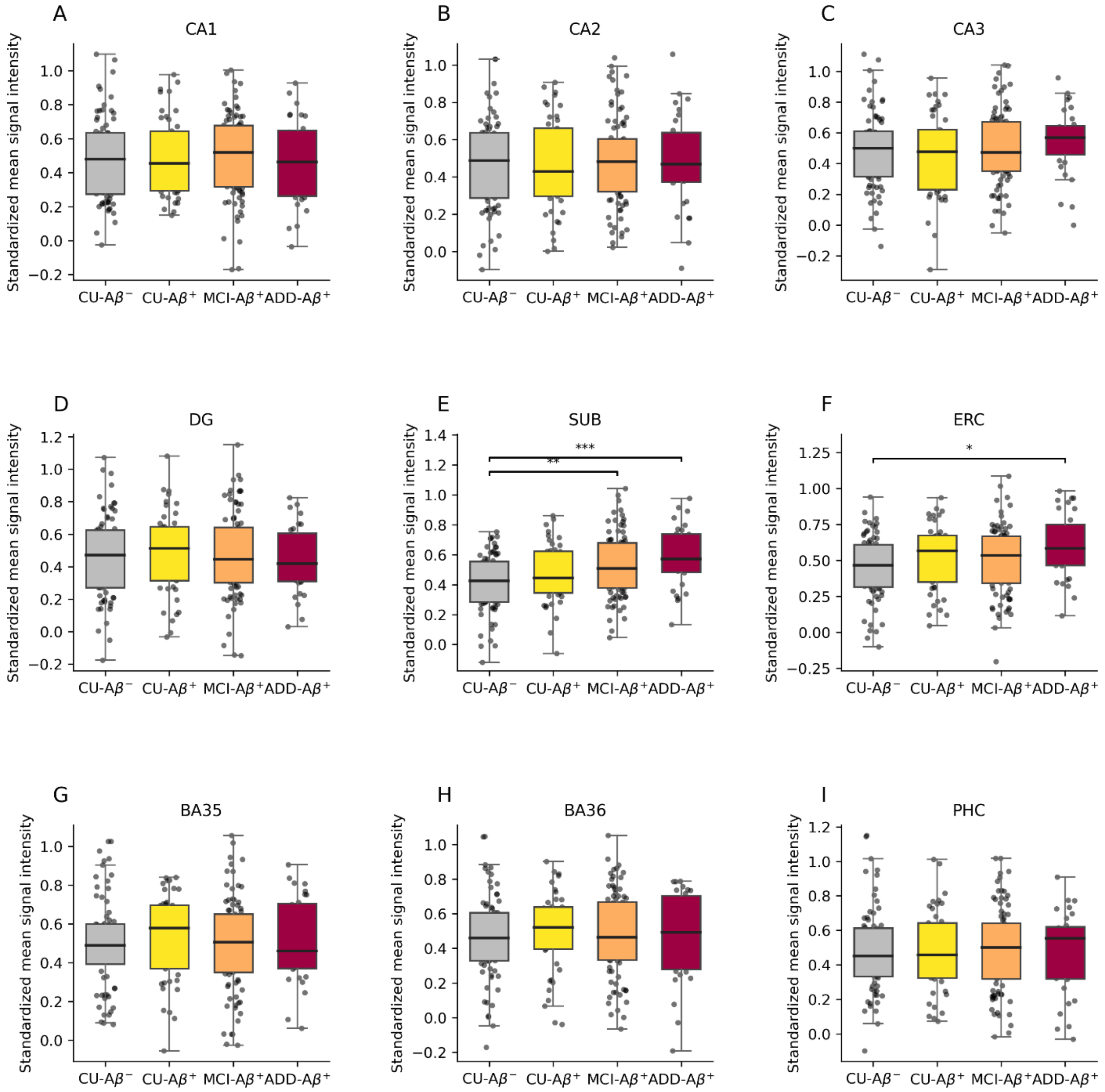
A
CA1
B
CA2
C
CA3
D
DG
E
SUB
F
ERC
G
BA35
H
BA36
I
PHC
Standardized mean signal intensity
CU-Aβ⁻ CU-Aβ⁺ MCI-Aβ⁺ ADD-Aβ⁺
**
***
*

Supplementary Fig. 5. Group-wise distributions of first-order entropy values in hippocampal subfields and medial temporal cortical regions. The mean first-order entropy value was calculated for each ROI—CA1 (A), CA2 (B), CA3 (C), DG (D), SUB (E), ERC (F), BA35 (G), BA36 (H), and PHC (I)—from the HighResHippo T2-weighted MRI. Group-wise comparisons were conducted using one-way analysis of variance with post hoc pairwise comparisons corrected for multiple testing using the Benjamini–Hochberg FDR procedure. The sample sizes were CU-Aβ^- (N = 76), CU-Aβ^+ (N = 45), MCI-Aβ^+ (N = 89), and ADD-Aβ^+ (N = 31). Each data point represents the first-order entropy from a single participant. All the ROIs show the downward shifts along the AD continuum, although not statistically significant. AD, Alzheimer's disease; ADD, Alzheimer's disease dementia; BA, Brodmann area; CA, cornu ammonis; DG, dentate gyrus; ERC, entorhinal cortex; FDR, false discovery rate; MTL, medial temporal lobe; PHC, parahippocampal cortex; SUB, subiculum.

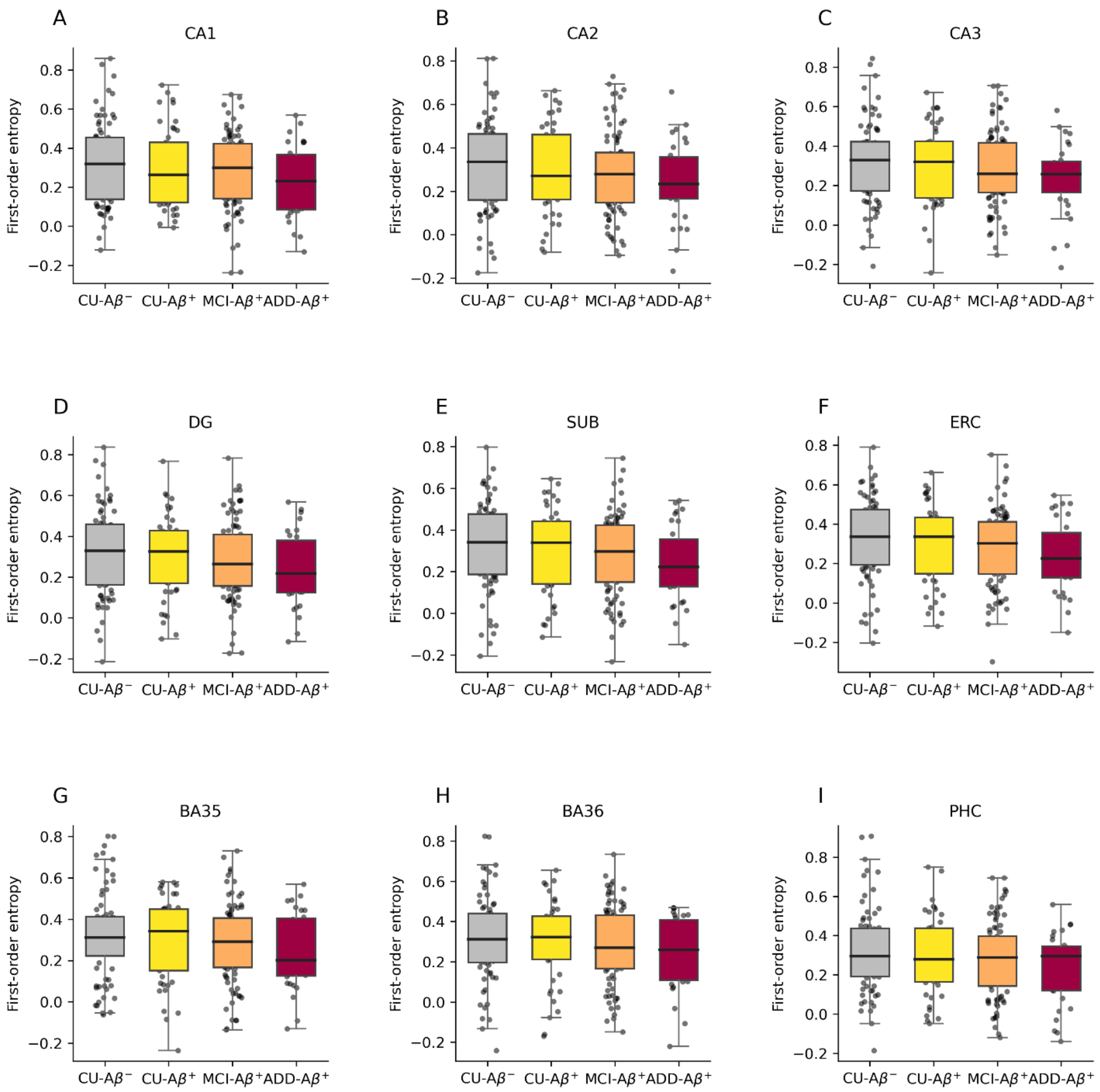
A
CA1
B
CA2
C
CA3
D
DG
E
SUB
F
ERC
G
BA35
H
BA36
I
PHC
First-order entropy
CU-Aβ⁻
CU-Aβ⁺
MCI-Aβ⁺
ADD-Aβ⁺

Supplementary Fig. 6. Group-wise histograms of subject-wise higher-order intensity moments in the subiculum. Skewness (A) and kurtosis (B) were calculated from the subiculum intensity histogram on HighResHippo T2-weighted MRI. Along the AD continuum (CU-Aβ^{-}, CU-Aβ^{+}, MCI-Aβ^{+}, ADD-Aβ^{+}), the 16-bin histograms display the distribution of these values. The sample sizes were CU-Aβ^{-} (N = 76), CU-Aβ^{+} (N = 45), MCI-Aβ^{+} (N = 89), and ADD-Aβ^{+} (N = 31). N represents the number of participants included in each group. Kurtosis was higher in Aβ^{+} groups than in CU-Aβ^{-} (3.42 ± 0.72), with the highest mean in CU-Aβ^{+} (3.88 ± 1.54). Skewness increased toward ADD-Aβ^{+} (0.15 ± 0.42), indicating progressively more peaked and slightly right-tailed intensity distributions in later stages.

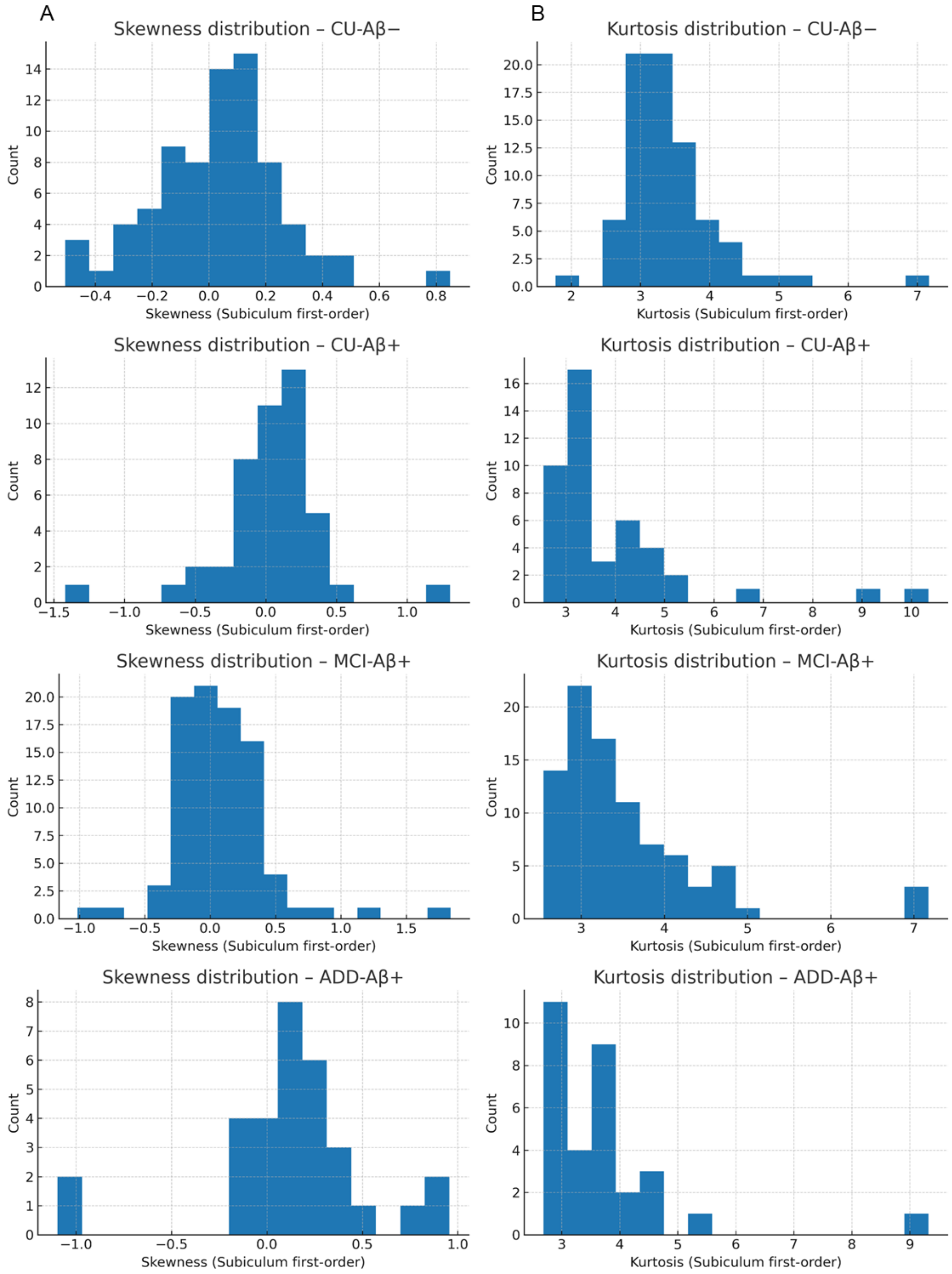
A
Skewness distribution – CU-Aβ−
Count
Skewness (Subiculum first-order)
B
Kurtosis distribution – CU-Aβ−
Count
Kurtosis (Subiculum first-order)
Skewness distribution – CU-Aβ+
Count
Skewness (Subiculum first-order)
Kurtosis distribution – CU-Aβ+
Count
Kurtosis (Subiculum first-order)
Skewness distribution – MCI-Aβ+
Count
Skewness (Subiculum first-order)
Kurtosis distribution – MCI-Aβ+
Count
Kurtosis (Subiculum first-order)
Skewness distribution – ADD-Aβ+
Count
Skewness (Subiculum first-order)
Kurtosis distribution – ADD-Aβ+
Count
Kurtosis (Subiculum first-order)

Supplementary Fig. 7. Representative image patches illustrating GLRLM-based run entropy. Representative coronal HighResHippo T2-weighted MRI image patches are shown from the left subiculum (red dashed boxes) for illustrative purposes. (A) A 70-year-old man, cognitively unimpaired and amyloid-β negative (CU–$A\beta^{-}$). The subicular texture appears relatively uniform, with longer and more continuous intensity runs, consistent with lower GLRLM-based run entropy. (B) A 67-year-old man with Alzheimer's disease dementia and amyloid-β positivity (ADD–$A\beta^{+}$). The subicular texture appears more heterogeneous, characterized by shorter and more irregular run-length patterns, consistent with higher GLRLM-based run entropy.

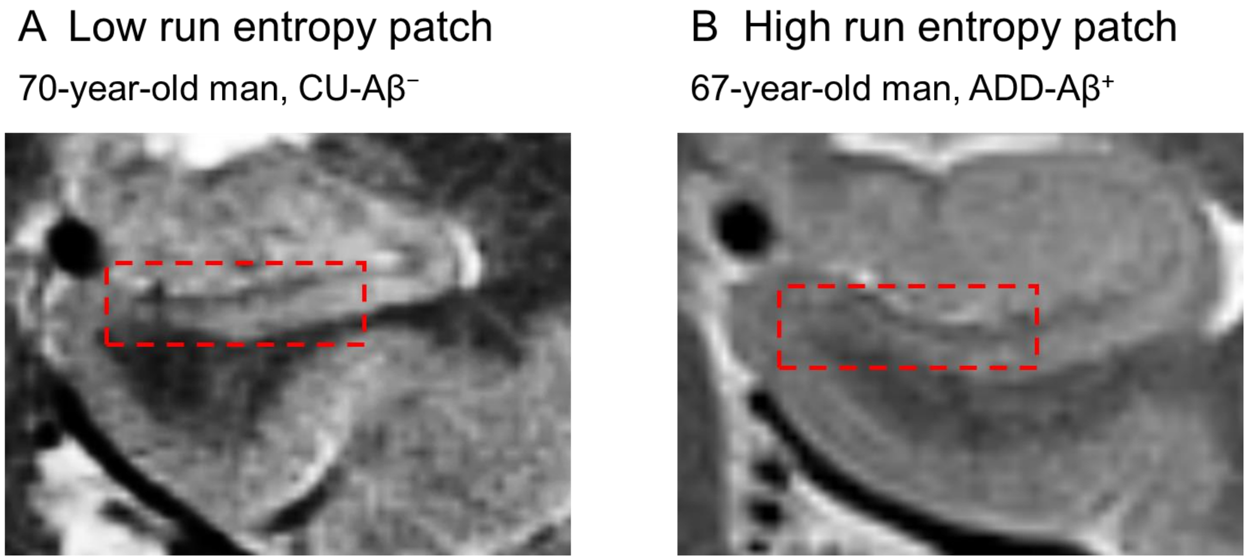

Supplementary Fig. 8. Residual diagnostics for the regression models of MMSE and MoCA vs. GLRLM-based run entropy. Histograms of residuals indicate mild right-skewness for MMSE (A) and approximately normal distributions for MoCA (B) and GLRLM-based run entropy (C). Residual plots are shown to assess model assumptions of linearity, homoscedasticity, and normality for the multiple linear regression analyses. The sample sizes for the regression analyses were N = 238 for the MMSE and N = 83 for the MoCA, reflecting the availability of cognitive data. Each data point represents an individual participant. Residuals-versus-fitted plots show no evidence of heteroscedasticity or systematic patterns, supporting the validity of model assumptions for both MMSE (D) and MoCA (E).

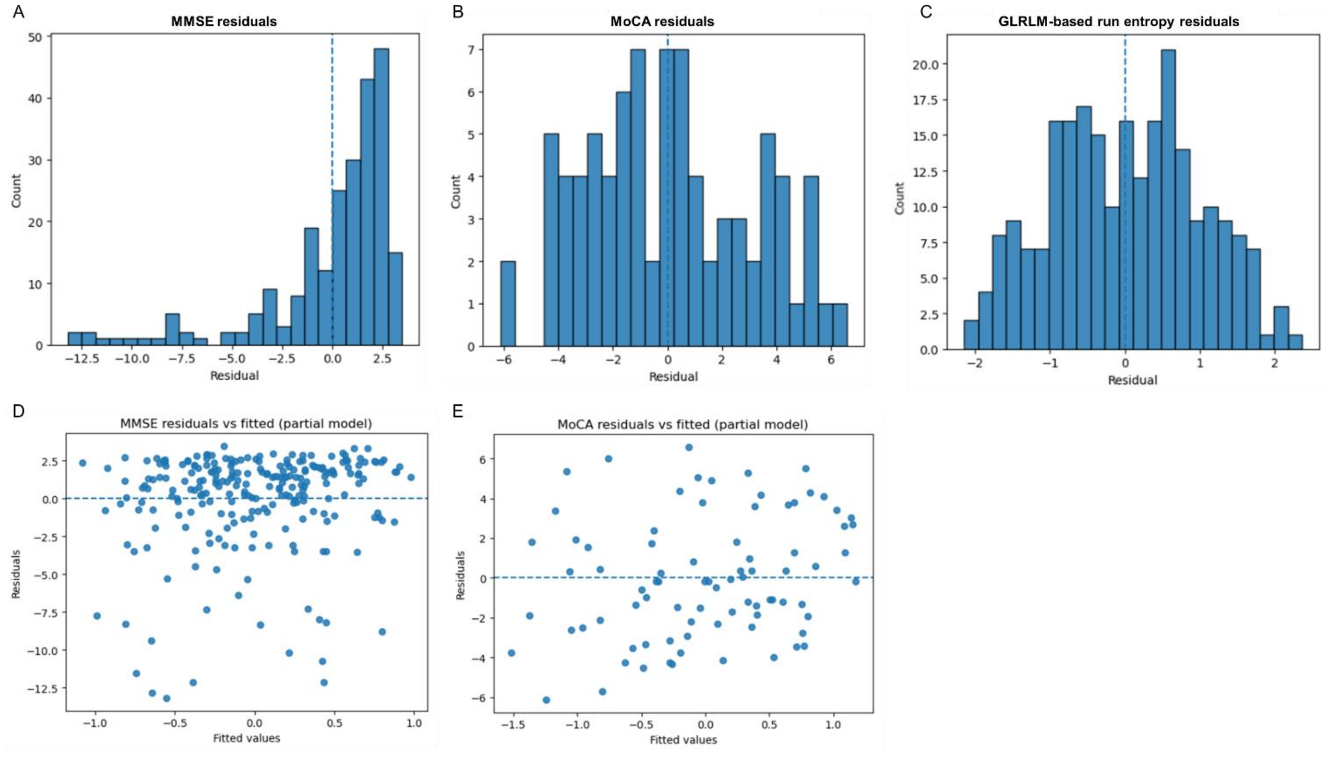

Supplementary Fig. 9. Associations between cerebrospinal fluid tau levels and texture features were evaluated using multiple linear regression models adjusted for age, sex, years of education, and APOE ε4 carrier status. The total sample size for all regression analyses was N = 241. Each data point represents an individual participant, plotted using covariate-adjusted (residualized) values of both tau levels and imaging features. Residualized CSF tau levels were significantly associated with residualized GLRLM-based run entropy values ([A] p-tau181: standard β = 1.341; 95% CI 0.103 to 2.580; FDR-corrected P = 0.034, [B] t-tau: standard β = 13.934; 95% CI 2.425 to 25.443; FDR-corrected P = 0.018), as well as those restricted to the SI direction ([C] p-tau181: standard β = 2.307; 95% CI 0.767 to 3.848; FDR-corrected P = 0.003, [D] t-tau: standard β = 18.822; 95% CI 4.698 to 32.947; FDR-corrected P = 0.009). FDR, false discovery rate; GLRLM, gray-level run-length matrix; p-tau181, phosphorylated tau at threonine 181; SI, superior–inferior; t-tau, total tau.

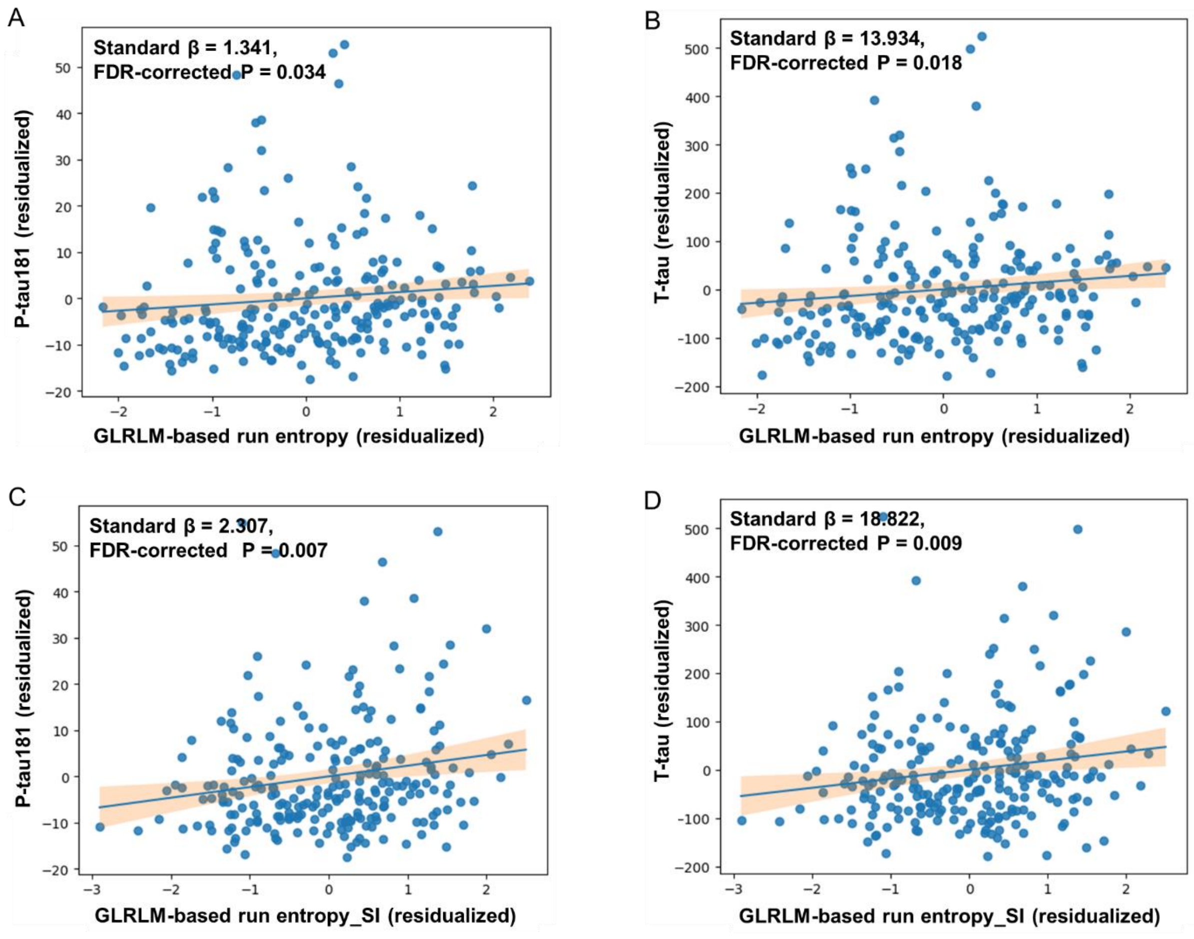

Supplementary Fig. 10. Residual diagnostics for the regression models of CSF tau levels vs. GLRLM-based run entropy. Histograms of residuals indicate approximately normal distributions for p-tau181 (A) and t-tau (B). Residual plots are shown to assess model assumptions of linearity, homoscedasticity, and normality for the multiple linear regression analyses. The total sample size for all regression analyses was N = 241. Each data point represents an individual participant. Residuals-versus-fitted plots show no evidence of heteroscedasticity or systematic patterns, supporting the validity of model assumptions for both p-tau181 (C) and t-tau (D).

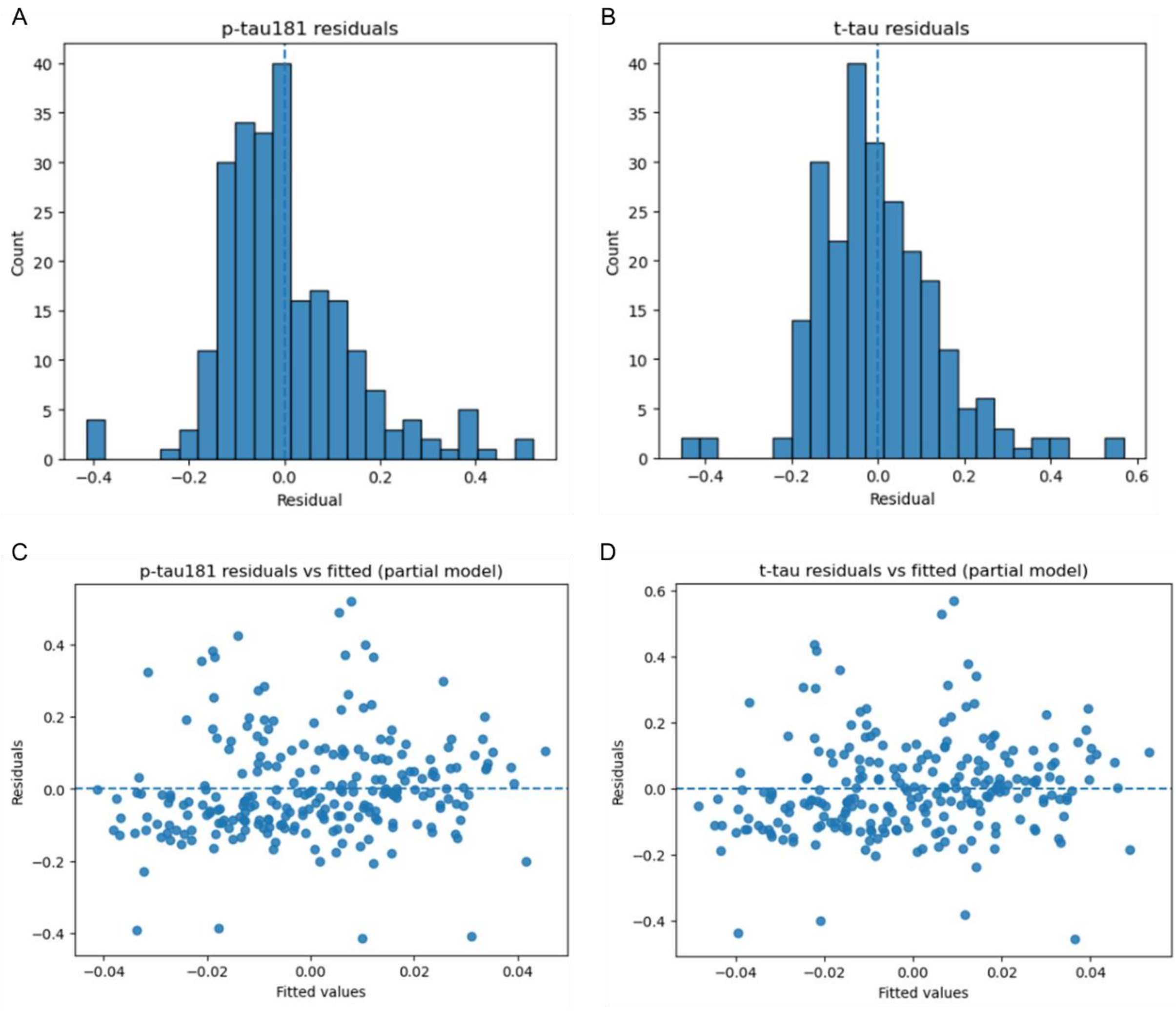

References

1. Bittner T, Zetterberg H, Teunissen CE, et al. Technical performance of a novel, fully automated electrochemiluminescence immunoassay for the quantitation of β-amyloid (1-42) in human cerebrospinal fluid. Alzheimers Dement 2016;12:517-526.

2. Hansson O, Seibyl J, Stomrud E, et al. CSF biomarkers of Alzheimer's disease concord with amyloid-β PET and predict clinical progression: A study of fully automated immunoassays in BioFINDER and ADNI cohorts. Alzheimers Dement 2018;14:1470-1481.

3. Jack CR, Jr., Bennett DA, Blennow K, et al. NIA-AA Research Framework: Toward a biological definition of Alzheimer's disease. Alzheimers Dement 2018;14:535-562.

4. Jack CR, Jr., Arani A, Borowski BJ, et al. Overview of ADNI MRI. Alzheimers Dement 2024;20:7350-7360.

5. Arani A, Borowski B, Felmlee J, et al. Design and validation of the ADNI MR protocol. Alzheimers Dement 2024;20:6615-6621.

6. Das SR, Avants BB, Pluta J, et al. Measuring longitudinal change in the hippocampal formation from in vivo high-resolution T2-weighted MRI. Neuroimage 2012;60:1266-1279.

7. Yushkevich PA, Pluta JB, Wang H, et al. Automated volumetry and regional thickness analysis of hippocampal subfields and medial temporal cortical structures in mild cognitive impairment. Hum Brain Mapp 2015;36:258-287.

8. Xie L, Wisse LEM, Pluta J, et al. Automated segmentation of medial temporal lobe subregions on in vivo T1-weighted MRI in early stages of Alzheimer's disease. Hum Brain Mapp 2019;40:3431-3451.

9. Canada KL, Mazloum-Farzaghi N, Rådman G, et al. A (sub)field guide to quality control in hippocampal subfield segmentation on high-resolution T(2)-weighted MRI. Hum Brain Mapp 2024;45:e70004.

10. Mayerhoefer ME, Materka A, Langs G, et al. Introduction to Radiomics. J Nucl Med

2020;61:488-495.

11. Wearn A, Raket LL, Collins DL, et al. Longitudinal changes in hippocampal texture from healthy aging to Alzheimer's disease. Brain Commun 2023;5:fcad195.

12. Yushkevich PA, Ittyerah R, Li Y, et al. Morphometry of medial temporal lobe subregions using high-resolution T2-weighted MRI in ADNI3: Why, how, and what's next? Alzheimers Dement 2024;20:8113-8128.
